# Interaction of *Cryptococcus neoformans* Rim101 and Protein Kinase A Regulates Capsule

**DOI:** 10.1371/journal.ppat.1000776

**Published:** 2010-02-19

**Authors:** Teresa R. O'Meara, Diana Norton, Michael S. Price, Christie Hay, Meredith F. Clements, Connie B. Nichols, J. Andrew Alspaugh

**Affiliations:** Departments of Medicine and Molecular Genetics/Microbiology, Duke University School of Medicine, Durham, North Carolina, United States of America; Carnegie Mellon University, United States of America

## Abstract

*Cryptococcus neoformans* is a prevalent human fungal pathogen that must survive within various tissues in order to establish a human infection. We have identified the *C. neoformans* Rim101 transcription factor, a highly conserved pH-response regulator in many fungal species. The *rim101Δ* mutant strain displays growth defects similar to other fungal species in the presence of alkaline pH, increased salt concentrations, and iron limitation. However, the *rim101Δ* strain is also characterized by a striking defect in capsule, an important virulence-associated phenotype. This capsular defect is likely due to alterations in polysaccharide attachment to the cell surface, not in polysaccharide biosynthesis. In contrast to many other *C. neoformans* capsule-defective strains, the *rim101Δ* mutant is hypervirulent in animal models of cryptococcosis. Whereas Rim101 activation in other fungal species occurs through the conserved Rim pathway, we demonstrate that *C. neoformans* Rim101 is also activated by the cAMP/PKA pathway. We report here that *C. neoformans* uses PKA and the Rim pathway to regulate the localization, activation, and processing of the Rim101 transcription factor. We also demonstrate specific host-relevant activating conditions for Rim101 cleavage, showing that *C. neoformans* has co-opted conserved signaling pathways to respond to the specific niche within the infected host. These results establish a novel mechanism for Rim101 activation and the integration of two conserved signaling cascades in response to host environmental conditions.

## Author Summary

*Cryptococcus neoformans* is an environmental fungus and an opportunistic human pathogen. Survival of this fungus within a human host depends on its ability to sense the host environment and respond with protective cellular changes. It is known that the cAMP/PKA signal transduction cascade is important for sensing host-specific environments and regulating the cellular adaptations, such as capsule and increased iron uptake, that are necessary for growth inside the infected host. Here we document that, unlike what has been described in other fungal species, a *C. neoformans* Rim101 homologue is directly regulated by PKA. The Rim101 signaling pathway is also involved in capsule regulation and virulence. Our study demonstrates that Rim101 integrates two conserved signal transduction cascades, and it is important in regulating microbial pathogenesis.

## Introduction

All cells, including pathogenic microorganisms, must be able to sense and respond to changes in their environment. As these cells enter a human host, they need to protect themselves from the immune system and rapidly adapt to human physiologic conditions, such as low nutrient availability, varying pH, and mammalian concentrations of carbon dioxide [1]. Therefore, they must coordinate multiple signaling pathways in order to control appropriate cellular responses.

One of the most common environmental stresses for pathogenic fungi is a change in the extracellular pH. Alterations in pH can affect a large number of cellular processes including membrane and cell wall stability, morphogenesis, protein stability and function, and nutrient uptake [2]–[8]. Many of these responses to pH are regulated by the Rim101 transcription factor and its homologues (PacC in filamentous fungi). Additionally, many pathogenic fungi respond to the neutral or slightly alkaline pH of the host by inducing virulence-associated phenotypes [2], [9]–[14]. Therefore, in diverse fungi such as *Aspergillus* and *Candida* species, mutants defective in pH sensing/response no longer induce phenotypes associated with virulence in pathogenic species. For example, *C. albicans rim101Δ/Δ* mutants do not undergo pH-dependent dimorphic switching, do not appropriately increase uptake of iron, and do not secrete the proteases and phosphatases necessary for invasion of host tissues [3], [5], [15]–[19]. *A. nidulans pacC (rim101)* mutants display decreased growth, decreased secondary metabolite production, and defective invasive growth [9], [14], [20]–[22]. Although *A. nidulans* is non-pathogenic, these cellular processes have been associated with virulence in other *Aspergillus* species.

In addition to the direct effects of ambient pH on cell integrity and various metabolic processes, pH changes also affect nutrient uptake. For example, under alkaline conditions, the availability of free iron is greatly reduced as the iron equilibrium shifts from the bioavailable ferrous form to the insoluble ferric form. Studying iron flux is an important new horizon in fungal pathogenesis, as the human host keeps free iron levels at extremely low concentrations (10^−18^M) through constitutively expressing iron-binding proteins such as transferrin and lactoferrin. In this way, the host protects against invading microorganisms. Fungal pathogens unable to increase iron uptake in this iron-limited host environment often have severe defects in virulence [5], [23]–[26]. The pH-responsive Rim101 transcription factor is involved in the regulation of iron homeostasis, directly binding to the promoters of genes encoding high affinity iron uptake proteins: iron transporters, iron permeases and siderophore transporters [20],[23],[27].

*Cryptococcus neoformans* is an opportunistic human fungal pathogen. Unlike the distantly related pathogens *Candida albicans* or *Aspergillus fumigatus, C. neoformans* grows within a very narrow range of pH values in the host. It grows well at the human physiological pH of the blood and cerebrospinal fluid (pH 7.4) as well as in acidic environments such as the phagolysosome of the macrophage (pH 5) [28],[29]. However, unlike *C. albicans* and *A. fumigatus, C. neoformans* demonstrates a severe growth defect above pH 8. Despite this increased sensitivity to alkaline pH, there is still evidence that *C. neoformans* responds to the slightly alkaline pH of the infected host blood by inducing virulence-associated phenotypes. Capsule production, a major virulence determinate, is optimal at human physiological pH [28], [30]–[32].

On a molecular level, *C. neoformans* capsule synthesis is transcriptionally regulated by elements of the cAMP/PKA pathway. Strains with mutations in core cAMP signaling elements (such as the Gpa1 Gα protein, adenylyl cyclase Cac1, or the PKA catalytic subunit Pka1) display defective expression of capsule, and these mutant strains are attenuated for virulence in animal models of cryptococcosis. When the regulatory subunit of PKA is mutated in the *pkr1Δ* strain, the cells have constitutive activation of PKA signaling and display high production of capsule, even in non-inducing conditions [33]–[38]. Other inducing conditions for capsule include iron deprivation, nutrient limitation, and the presence of serum. The mechanisms by which these environmental signals are sensed and subsequently transduced to specific intracellular signaling pathways are not yet known. To explore the interaction of the inducing environmental signals, signal transduction pathways, and downstream effectors controlling capsule synthesis, we have begun to characterize specific transcription factors that are predicted to be targeted by the cAMP pathway, and which also directly control capsule gene expression. By examining the biological function and regulation of the *C. neoformans* Rim101 homolog transcription factor, we have determined a new pathway for *C. neoformans* regulation of capsule production. We have also defined a novel interaction between the highly conserved Rim and PKA signaling pathways.

## Results

### Identifying PKA-regulated transcription factors

To identify potential transcriptional regulators of *C. neoformans* capsule that are also directly phosphorylated by PKA, we used a bioinformatic survey of the annotated *C. neoformans* genome. We first searched the available annotation (GO terms, gene names, homology designators) for proteins likely to be involved in transcriptional regulation: transcription factors, DNA binding motifs, zinc finger domains, and other transcriptional regulators. Given the incomplete annotation of the genome, we accepted that many transcriptional regulators might be initially misidentified or excluded by this approach.

Among this subset of proteins, we searched the predicted protein sequence for consensus sequences for PKA phosphorylation (R/K R/K X S/T), to identify potential direct targets of the Protein Kinase A enzyme. One of these proteins is a homologue of Rim101/PacC, a conserved fungal C2H2 transcription factor (GenBank ID CNH00970). The *C. neoformans* Rim101 protein contains a RRASSL motif at aa730, and has highest homology to *Aspergillus nidulans* PacC in the C2H2 domain. Analysis of the protein for conserved domains revealed that the only significant Pfam-A match is the zinc finger C2H2 domain (aa133–155).

### Disruption of *RIM101*

To characterize the biological role of this *C. neoformans* transcriptional regulator, we disrupted the *C. neoformans RIM101* gene by homologous recombination. Southern blot analysis confirmed that the *rim101::nat* mutant allele precisely replaced the native gene in the *rim101Δ* deletion strain (TOC2) (Table 1) without additional ectopic integration events (data not shown). In addition to this mutant strain in which the entire *RIM101* open reading frame was deleted, we created an independent *rim101Δ* mutant with a partial gene deletion. All phenotypes between these strains were identical, and the TOC2 strain was therefore chosen as the representative *rim101Δ* mutant strain. To ensure that any phenotypes observed in the *rim101Δ* mutant strain were due to disruption of the *RIM101* gene, we created a *rim101Δ* *+RIM101* complemented strain (TOC4) by integrating a wild-type copy of the *RIM101* gene into the genome of the *rim101Δ* mutant strain.

**Table 1:** List of strains used in this study.

Name	Genotype	Reference
H99	*Wild-type*	[90]
TOC 2	*rim101::nat*	this paper
TOC 4	*rim101::nat RIM101-neo*	this paper
TOC 10	*rim101::nat + pHIS-Gfp-Rim101-neo*	this paper
TOC 12	*ura5 pka1::URA5 + pHIS-Gfp-Rim101-neo*	this paper
TOC 13	*ura5 pkr1::URA5 + pHIS-Gfp-Rim101-neo*	this paper
TOC 17	*rim20::nat*	this paper
TOC 21	*rim20::nat + pHIS-Gfp-Rim101-neo*	this paper
TOC 18	*rim101::nat + pHIS-Gfp-Rim101-S773A-neo*	this paper
TOC 20	*ura5 pkr1::URA5 + pHIS-Gfp-Rim101-S773A-neo*	this paper
TOC 22	*rim20::nat: + pHIS-Gfp-Rim101-S773A-neo*	this paper
JKH 7	*ura5 pka1::URA5*	[91]
CDC 7	*ura5 pkr1::URA5*	[36]
TYCC 33	*ade2 cap59::ADE*	[92]

The *rim101Δ* mutant strain grew at a similar rate as wild-type on rich media (YPD) and minimal media (YNB) at 30°C, 37°C and 39°C. We found no defect in melanin production on Niger-seed medium. We also examined the mutant strain for resistance to hydrogen peroxide and paraquat by disc diffusion assays and established that the zone of inhibition for the *rim101Δ* mutant strain was similar to that of wild-type, showing no additional sensitivity to reactive oxygen species.

### Rim101 is required for capsule induction

**Figure 1:**
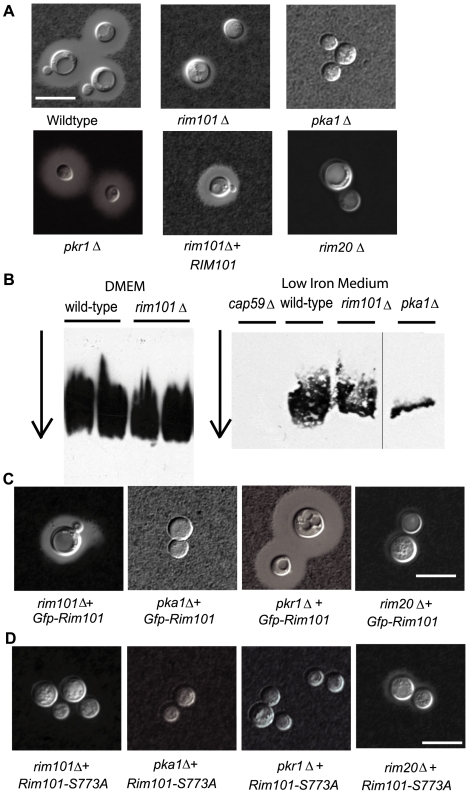
*C. neoformans* Rim101 is required for capsule attachment. A. *rim101Δ* mutants have a capsule defect. Cells were incubated in capsule inducing conditions for 3 days. Capsule was assessed by staining with India ink and visualizing the zone of exclusion at 63× magnification (scale bar = 10 m). B. The *rim101Δ* mutant sheds equivalent capsule to wild-type. Electrophoretic mobility and quantity of shed polysaccharide was assessed by a blotting technique of culture medium filtrate, using an anti-GXM antibody to probe for capsule as previously described [47]. Cells were incubated in Dulbecco's modified Eagle's medium or low iron medium for 1 week before filtering. Arrow indicates direction of electrophoresis. C. Gfp-tagged Rim101 is functional. Cells were incubated in capsule inducing conditions for 3 days. Capsule was assessed by staining with India ink and visualizing the zone of exclusion at 63× magnification (scale bar = 10 m). D. Rim101-S773A does not complement capsule. Cells were incubated in capsule inducing conditions for 3 days. Capsule was assessed by staining with India ink and visualizing the zone of exclusion at 63× magnification (scale bar = 10 m).

The *rim101Δ* mutant strain has a major defect in polysaccharide capsule, an important virulence factor in *C. neoformans*. We incubated wild-type and *rim101Δ* mutant strains in a capsule inducing medium, Dulbecco's modified Eagle's medium (DMEM) containing 25 mM NaHCO_3_, for 72 hrs at 37°C and 5% CO_2_. Incubation in this media usually leads to large polysaccharide capsules surrounding each cell which can be quantitatively measured by analyzing the percent packed cell volume (Cryptocrit analysis) [31]. The *rim101Δ* mutant strain exhibits markedly reduced capsule around the cell (3.6% packed cell volume) compared to the wild-type strain (6.2% packed cell volume). The *rim101Δ* mutant capsule-defective phenotype was not noted in previous reports of the *C. neoformans* Rim101 protein [39]; therefore, we confirmed our observation in several ways. We examined several independent *rim101Δ* mutants, including partial and complete gene deletions, which all displayed similar capsule defects in the inducing conditions. These differences in capsule were microscopically visualized by the exclusion of India ink (Figure 1A). Reintroduction of the wild-type allele fully complemented the capsule phenotype (6.8% packed cell volume).

To confirm the role of Rim101 and the Rim pathway in *C. neoformans* capsule production, we searched the *C. neoformans* genome for conserved elements of the Rim pathway, and we identified the *RIM20* gene. Rim20 is a scaffold protein required for Rim101 cleavage/activation in other fungal species [6], [40]–[45]. When we mutated this gene, we observed a similar capsule defect in the *rim20Δ* mutant compared to the *rim101Δ* mutant strain (Figure 1A).

*C. neoformans* capsule is secreted out of the cell and subsequently bound to the cell wall [46]. To determine whether *rim101Δ* mutant strains produce and secrete capsular polysaccharide, we used a previously described gel electrophoresis technique to quantify this polymer [47]. *C. neoformans* cells were incubated in capsule-inducing DMEM for 1 week, after which polysaccharide that was shed into the medium was analyzed for relative abundance and size by reactivity against an anti-GXM antibody (mAb18B7). We noted a similar amount of secreted polysaccharide in the *rim101Δ* mutant as wild-type (Figure 1B) suggesting that there is no significant GXM synthesis defect in the *rim101Δ* strain. We repeated this assay using low iron medium [31] as the capsule inducing condition instead of Dulbecco's modified Eagle's medium, and we again observed a similar amount of secreted capsule in wild-type and *rim101Δ* mutant strains (Figure 1B). As previously described, we used a *cap59* mutant strain that is unable to secrete capsule as a negative control [48] and detected no capsule by this assay in this strain. We also demonstrated the quantitative nature of this assay by analyzing the *pka1Δ* mutant strain, in which there is a previously documented decrease in capsule production compared to wild-type [36] (Figure 1B). Therefore, the hypocapsular phenotype of the *rim101Δ* mutant strain in the presence of intact polysaccharide production suggests a defect in capsule attachment. This result is similar to the phenotype of the *C. neoformans ags1Δ* mutant strain which can synthesize and secrete capsule but cannot bind it [49],[50].

### CnRim101 retains conserved physiological roles with Rim101/PacC from other fungal species

In fungi as diverse as *Aspergillus, Saccharomyces, Candida,* and *Ustilago* species, the Rim101/PacC proteins control multiple pH-related phenotypes, including regulating iron homeostasis; maintaining membrane and cell wall-associated proteins; and secreting proteases, secondary metabolites, and phosphatases [2],[5],[14],[51]. Many of these factors are important in the virulence of pathogenic fungi.

**Figure 2:**
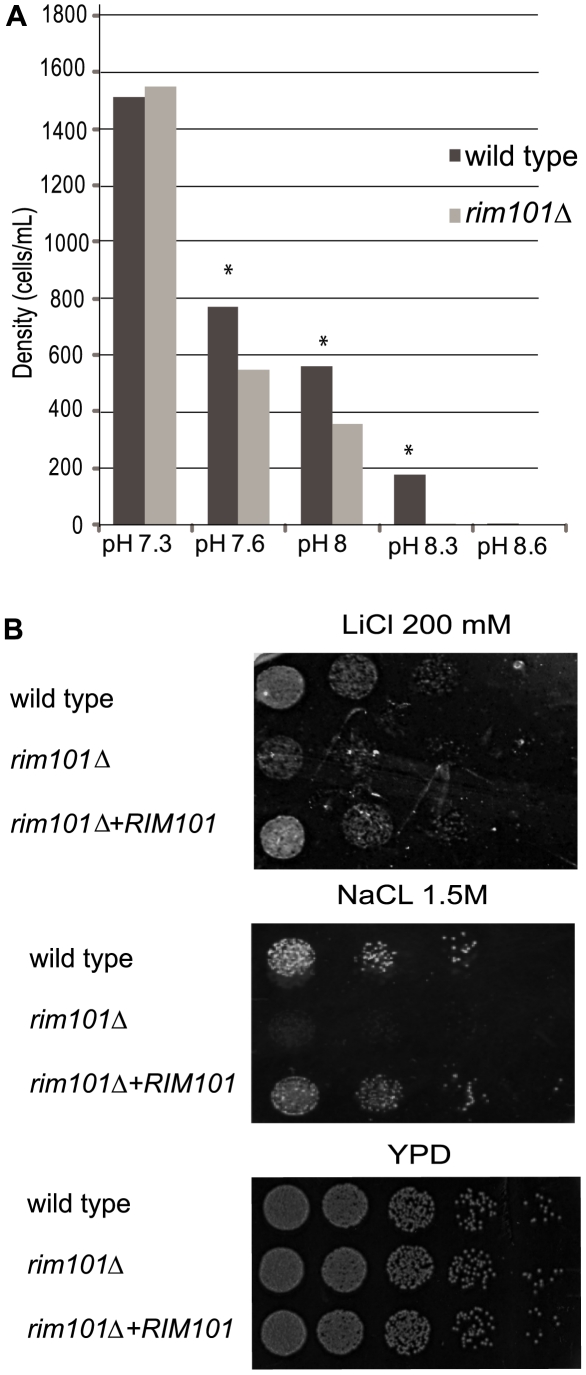
Rim101 retains conserved phenotypes from other fungal species. A. The *rim101Δ *mutant is sensitive to alkaline pH. Cells were incubated in buffered YNB, and growth was determined by cell counts after 72 hours. B. The *rim101Δ* mutant is sensitive to salt stress. 1×10^5^ cells from each strain were serially diluted (5-fold dilution) onto YPD plates containing 1.5M NaCl or 200 mM LiCl. The plates were incubated at 30°C for 3 days. Cells were plated onto YPD plates for 48 hours as a control.

We tested the *C. neoformans rim101Δ* mutant strain to determine if this protein retains conserved physiological roles with Rim101/PacC proteins in other fungal species. On alkaline media, the *rim101Δ* mutant strain exhibits a severe growth defect compared to the wild-type and the reconstituted strains at alkaline pH above 7.6 (*p*<0.01) (Figure 2A). Similar to other *rim101-*defective fungal strains, the *C. neoformans rim101Δ* mutant also displayed sensitivity to media containing 200 mM LiCl or 1.5M NaCl (Figure 2B). To confirm that this growth defect was due to specific sensitivity to ionic stress as opposed to general sensitivity to osmotic stress, we tested the cells for growth on media containing 2.5M sorbitol and detected no difference in growth between the *rim101Δ* mutant strain and wild-type. We also determined no defects in response to cell wall stress for the *rim101Δ* mutant strain during growth on calcofluor white, Congo red, or 0.05% SDS. These data suggests that, unlike in other fungal species, there are no major defects in cell wall integrity in the *C. neoformans rim101Δ* mutant strain.

### Rim101 localization is regulated by PKA and Rim20

In other fungal species, the Rim101 protein is activated by cleavage and subsequently localized to the nucleus, as expected for a transcription factor [52]. To test whether *C. neoformans* Rim101 is cleaved and localized to the nucleus, we created a Gfp-Rim101 fusion protein. We fused the green fluorescent protein gene to the N-terminus of the *RIM101* gene, expressing the new transgene under control of a constitutive histone promoter. Introduction of this plasmid (pTO2) by biolistic transformation into the *rim101Δ* mutant strains fully complemented the *rim101Δ* mutant capsule phenotype, indicating that the Gfp-Rim101 fusion protein is functional (Figure 1C).

**Figure 3:**
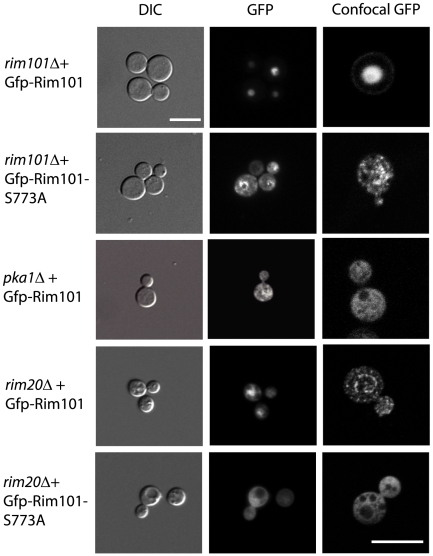
Rim101 localization in mutant backgrounds. Rim101 localization is dependent on PKA and Rim20. The pattern of Gfp-Rim101 localization in the indicated strains was visualized by DIC and fluorescent microscopy at 63× magnification and by confocal microscopy at 100× magnification.

Unlike *A. nidulans,* in which localization is dependent on activation, *C. neoformans* Rim101 is nuclear under all conditions tested. Using epifluorescent microscopy, we observed a nuclear pattern of localization for *C. neoformans* Gfp-Rim101 after 24 hours growth in various conditions, including YNB buffered at pH 8, Dulbecco's modified Eagle's medium, YNB (pH 5.4), and YPD (Figure 3). In contrast, the Gfp-Rim101 protein in the *pka1Δ* and *rim20Δ* mutant backgrounds localized to both the nucleus and the cytoplasm under the same growth conditions, suggesting that PKA activity and Rim20-mediated cleavage are both important for complete nuclear localization (Figure 3). In addition, overexpression of the Gfp-tagged Rim101 protein was not able to suppress the *pka1Δ* or the *rim20Δ* mutant capsule phenotype (Figure 1C).

To further explore the potential association of PKA and Rim101, we mutated the single Rim101 consensus sequence for PKA phosphorylation by changing serine 773 to an alanine, creating the Rim101-S773A mutant protein encoded in plasmid pTO3. When examining the localization of the Gfp-Rim101-S773A protein, we observed both nuclear and cytoplasmic fluorescence, similar to the localization of wild-type Gfp-Rim101 expressed in the *pka1Δ* and *rim20Δ* mutant backgrounds. Introduction of the Gfp-Rim101-S773A protein into the *rim20Δ* background also resulted in both nuclear and cytoplasmic localization of Rim10. Additionally, the Rim101-S773A mutant protein in the *rim101Δ* mutant background did not complement the capsule phenotype (Figure 1D). This observation, coupled with our documentation that some Gfp-Rim101-S773A protein is localizing to the nucleus, indicates that serine 773 is necessary for full function of the Rim101 protein. When we transformed the Rim101-S773A mutant protein into the *pkr1Δ* mutant strain, in which PKA signaling and capsule production are constitutively active, multiple independent transformants had markedly attenuated capsule, even though the wild-type *RIM101* gene was still present in these strains (Figure 1D). In contrast, introduction of the plasmid containing the wild-type Rim101 fused to Gfp into the *pkr1Δ* strain had no effect on capsule. This suggests that the Rim101-S773A mutant protein is acting in a dominant negative manner on *C. neoformans* capsule.

### Rim101 cleavage is mediated by PKA and Rim20

**Figure 4:**
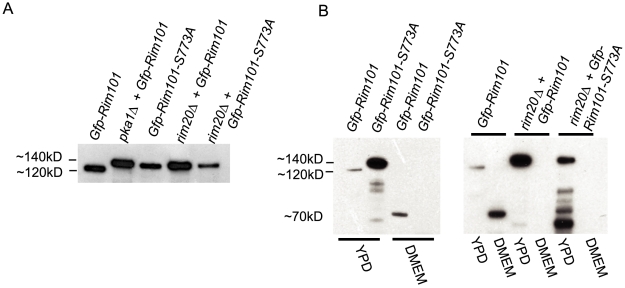
Western blot analysis of Rim101 in *rim101* and *pka1Δ* *mutant* backgrounds. A. Rim101cleavage is dependent on PKA and Rim20. Immunoprecipitated Gfp-Rim101 from *rim101Δ*+ *Gfp-rim101, rim101Δ* *+ Gfp-rim101-S773A, pka1Δ* *+ Gfp-rim10*, *rim20Δ* *+ Gfp-rim101*, and *rim20Δ* *+ Gfp-rim101-S773A* strains was immunoblotted using anti-GFP antibody. B. Rim101 is cleaved after induction in capsule media. Gfp-Rim101 was immunoprecipitated from *rim101Δ*, *rim101Δ* *+ rim101-S773A, rim20Δ* *+ Gfp-rim101*, and *rim20Δ* *+ Gfp-rim101-S773A* cell lysates after incubation in either YPD medium or the capsule-inducing medium DMEM at 30°C to mid-log phase. Samples were run on Bis-Tris gels and immunoblotted using an anti-GFP antibody.

To examine the interaction of PKA and the *C. neoformans* Rim pathway, we used western blotting techniques to compare Rim101 protein processing in multiple strain backgrounds. Using an anti-Gfp monoclonal antibody for detection, we identified bands corresponding to the Gfp-Rim101 fusion protein from cell lysates of cultures incubated in YPD to mid-log phase (Figure 4A). The protein we detected when both Pka1 and Rim20 were wild-type had a molecular weight of approximately 120kD (Figure 4A). In contrast, expression of the identical Gfp-Rim101 fusion protein in the *pka1* or *rim20Δ* mutant backgrounds resulted in a protein band of approximately 140kD, which is the predicted size of the full length fusion protein. The Gfp-Rim101-S773A protein also migrated with a reduced electrophoretic mobility in the *rim101Δ* or *rim20Δ* backgrounds, resulting in an approximately 140kD band. The 120kD processed form of Gfp-Rim101 was dependent on both PKA and Rim20. Treatment with lambda phosphatases did not alter the mobility of any of these bands (data not shown). We also observed multiple smaller bands in the *pka1Δ* and *rim20Δ* mutant backgrounds. These may represent degradation products, suggesting that both Pka1 and Rim20 are necessary to prevent aberrant proteosomal involvement [53]. In addition, the strains with a 140kD Gfp-Rim101 protein were the same strains that had both cytoplasmic and nuclear patterns of Gfp fluorescence. Only the 120kD processed form had predominantly nuclear localization (Figure 3).

*A. nidulans* PacC undergoes two successive cleavage events that regulate its function as an alkaline-responsive transcription factor [42],[43],[53],[54]. To examine whether *C. neoformans* Rim101 also undergoes a second cleavage event under activating conditions, we incubated the *rim101Δ* +*Gfp-RIM101* strain to mid-log phase in either YPD or capsule inducing media. When incubated in DMEM, we detected an additional band at approximately 70kD, corresponding to a potentially cleaved N-terminal fragment of the Rim101 protein (Figure 4B). This band was not present when PKA, Rim20, or the PKA phosphorylation consensus sequence were mutated, or under non-inducing conditions, demonstrating that PKA phosphorylation and Rim20 are necessary for this further cleavage of Rim101 under inducing conditions.

### Rim101 downstream targets—capsule and iron

To define the downstream targets of the Rim101 transcription factor, we performed comparative transcriptional profiling between the *rim101Δ* mutant strain and wild-type using whole genome microarrays. We confirmed these results for several genes using quantitative real-time PCR (data not shown). These results indicate that Rim101 controls the transcription of many genes involved in several categories of cellular function (Table 2).

**Table 2:** Subset of Rim101-dependent gene expression in capsule-inducing conditions.

Category	Gene ID	Description^a^	Fold change (WT/*rim101*)
**Metal homeostasis**			
	CNC01660	cytokine inducing-glycoprotein *CIG1*	471.88
	CNE04530	siderochrome-iron transporter *SIT1*	81.14
	CNM02420	acidic laccase, putative *FET3*	20.75
	CNM02430	ferric permease *CFT1*	16.59
	CNG00950	metalloreductase	16.09
	CND01080	copper uptake transporter *CTR4*	7.09
**Cell Wall**			
	CNA07540	mannoprotein *MP88*	−5.98
	CND03490	mannoprotein *MP98*	−6.52
	CNG04420	alpha-1,3-glucan synthase, *AGS1*	−3.78
	CNN00660	glucan 1,3 beta-glucosidase protein	−2.07
**Capsule biosynthesis**			
	CNH00170	phosphomannomutase *PMM1*	3.19
	CNL06460	UDP-glucose 6-dehydrogenase *UGD1*	2.87
	CNF00920	mannosyltransferase *CMT1*	−2.97
**Other**			
	CNA05130	sodium efflux pump *ENA1*	6.92

This list contains functionally annotated genes whose expression was at least 2-fold different in wild-type vs. *rim101Δ* mutant strain after incubation in capsule-inducing media for 3 hrs. Complete data set of all genes differentially regulated in the *rim101Δ* mutant strain can be found in Table S1.

a. Annotations obtained from NCBI database (http://www.ncbi.nlm.nih.gov/) and BLAST searches (http://www.ncbi.nlm.nih.gov/blast/) with additional hand editing.

Under capsule-inducing conditions, we were able to document differential expression for a limited number of genes that may be involved in capsule biosynthesis. We observed a 2.9-fold greater expression of *UGD1* in the wild-type compared to the *rim101Δ* mutant strain. *UGD1* encodes a UDP-glucose dehydrogenase that is necessary for UPD-glucuronic acid synthesis and thus capsule biosynthesis [55],[56]. A mannosyltransferase (Cmt1) was 2.97-fold greater expressed in the *rim101Δ* mutant strain than the wild-type. Mannosyltransferases such as Cmt1 have been implicated in the biosynthesis of GXM [57]. In addition, we observed a 3.2-fold decrease in expression of a phosphomannomutase gene (*PMM*) in the *rim101Δ* mutant strain. PMM is involved in the biosynthesis of GDP-mannose, another nucleotide sugar essential for capsule production, and is transcriptionally regulated by PKA [24], [58]–[61]. The data also show a small number of other nucleotide sugar-related genes that are differentially expressed and may be involved in capsule production. The fact that many highly inducible capsule genes are not transcriptionally regulated by Rim101 is consistent with our observation that the capsule defect is due to adherence, not production.

**Figure 5:**
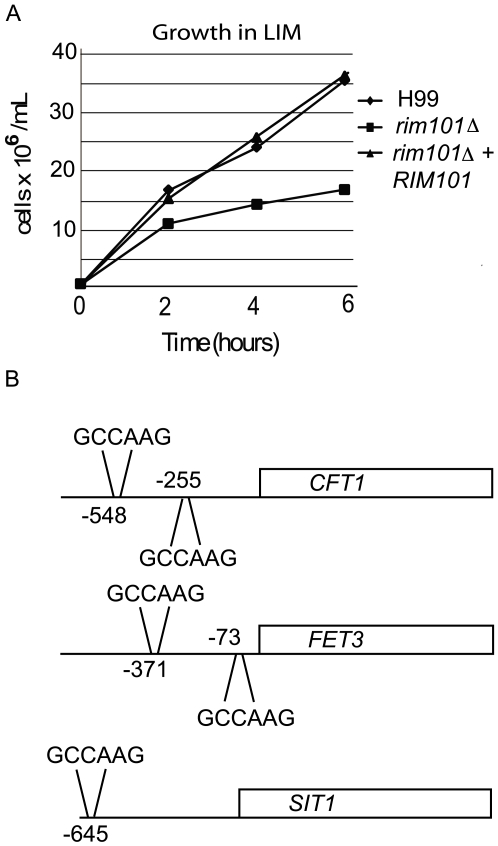
Rim101 in low iron media. A. Rim101 is required for growth in low-iron medium. Strains were incubated in low-iron medium and growth was quantified by monitoring the absorbance of the culture at 600 nm. B. The 5′-untranslated regions of *C. neoformans* genes involved in iron regulation were evaluated for the presence of putative Rim101 binding sites (GCCAAG or CCAAGAA).

Transcriptional profiling also suggested that Rim101 controls the expression of several genes involved in iron or metal homeostasis, including the iron transporter gene *CFT1*, siderophore importer gene *SIT1,* and copper transporter gene *CTR4* [24], [60]–[62]. In addition, we documented Rim101-dependent expression of homologues of *S. cerevisiae* iron permeases (*FRP1*) and reductases (*FET3*), which are known to be regulated by PKA and by the *Cryptococcus* transcription factor Cir1 [60],[61],[63],[64]. To demonstrate that decreased expression of these genes was biologically relevant, we incubated the *rim101Δ* mutant strain in low iron medium, and we observed a distinct growth defect compared to wild-type or the reconstituted *RIM101* strain (Figure 5A). *C. neoformans* strains typically induce capsule in response to growth in this medium. Although the *rim101Δ* mutant strain grew slowly in low iron media, it eventually reached saturation phase. However, even when grown to saturation, the *rim101Δ* mutant strain did not exhibit capsule in low iron media (data not shown). Further analysis of these iron homeostasis genes revealed that the promoters of all of these iron regulating genes contain the potential Rim101 consensus binding sequence GCCAAG or the diverged sequence CCAAGAA, recognized by the *S. cerevisiae, C. albicans* and *A. nidulans* Rim101 orthologs [7],[27],[65]. These results indicate that *C. neoformans* Rim101 retains conserved roles in regulating iron homeostasis and import.

### Rim101 and virulence

**Figure 6:**
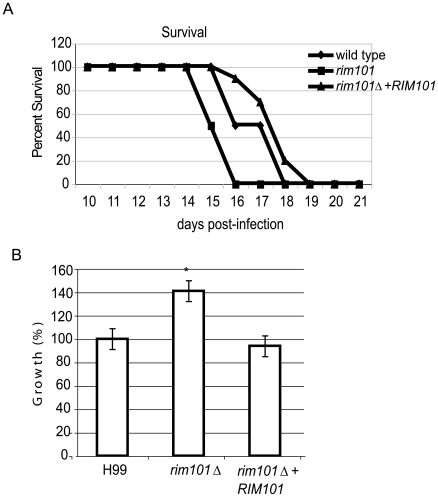
Virulence analysis of the *rim101Δ* *mutant* strain. A. *rim101Δ* *mutant* strains are hypervirulent in the murine inhalation model of cryptococcosis. AJc/r mice were inoculated intranasally with 5×10^5^cryptococcal cells and monitored for survival. B. *rim101Δ* *mutant* cells survive better than wild-type within macrophages. The *rim101Δ* mutant and isogenic wild-type strains were co-incubated with J744.1 macrophage-like cells previously activated by INF-gamma and LPS. Extracellular yeast cells were removed after one hour of co-incubation. After 24 hours of co-culture, the macrophages were lysed with SDS, and surviving yeast cells were quantitatively cultured. To precisely control for the number of added cells, the colony-forming units from each strain were normalized to that of wild-type cells. Data points represent the average of triplicate samples +/− standard error.

The ability to produce capsule is important for the pathogenicity of *C. neoformans*, and other capsule-deficient strains are severely attenuated for virulence in animal models of cryptococcosis [33], [35], [66]–[68]. In addition, the ability to obtain iron from the host and to grow in low iron conditions is important for microbial survival in the host [60],[63],[69]. We therefore hypothesized that the hypocapsular *rim101Δ* *mutant* would be avirulent in animal models of cryptococcosis. However, a recent manuscript in which the investigators tested virulence properties in a large collection of *C. neoformans* mutants suggested that the *rim101Δ* mutant strain might be more virulent than wild-type [39]. We therefore tested the role of Rim101 in *C. neoformans* pathogenicity. Female A/Jcr mice (10 per strain) were inoculated intranasally with 5×10^5^ CFU of the wildtype, *rim101Δ* mutant, or *rim101Δ* *+RIM101* complemented strains. Mice were monitored for survival and sacrificed at predetermined clinical endpoints predicting mortality (Figure 6A). Infection with either the wild-type or the *rim101Δ* *+RIM101* complemented strain resulted in complete mortality 18 and 19 days after infection respectively; there was no statistically significant difference between the survival of these two groups (*p* = 0.13). Mice infected with the *rim101Δ* mutant strain succumbed to the infection 16 days post-infection; this represents a statistically significant decrease in survival compared to animals infected with the wild-type strain (*p*<0.002). We repeated the infection and sacrificed mice on day 2, day 9 and day 14 to determine fungal burden in the lungs, spleen, and brain. In all organs, we found no statistically significant difference in rates of dissemination among the 3 inoculated strains.

The subtle but reproducible increased virulence of the *rim101Δ* mutant cells in the inhalation model of *C. neoformans* infection may be due to enhanced survival in the acidic environment of the alveolar macrophage. We specifically tested intracellular survival of the wild-type, *rim101Δ* mutant, and *rim101Δ* *+RIM101* complemented strains. As described previously, we co-cultured *C. neoformans* strains with IFNγ- and LPS-activated J774.1 macrophage-like cells [70]. There was no significant difference in the phagocytosis index of these strains by the macrophages, signifying that the altered capsule in the *rim101Δ* mutant did not affect fungal cell uptake into macrophages [71]. In contrast, the *rim101Δ* mutant cells demonstrated increased intracellular survival (*p*<0.004) within macrophages when normalized against the wild-type (Figure 6B).

## Discussion

Microbial pathogens use varied adaptive mechanisms to survive the harsh conditions of the infected host. *Cryptococcus neoformans* creates a polysaccharide capsule in response to host conditions such as low iron and high CO_2_ concentrations [1],[24]. The *C. neoformans* genome contains a number of genes involved in the biosynthesis of this capsule, and many of these genes are highly transcriptionally regulated, at least partially in response to the PKA pathway [38]. This led us to screen through the genome for transcription factors that are potentially regulated by PKA, and we previously found that the Nrg1 protein regulates capsule. Deletion of the *NRG1* gene resulted in a partial capsule reduction, and mutation of the putative PKA phosphorylation consensus sequence prevented full capsule induction. However, not all of the transcriptionally regulated capsule genes appeared to be targets for Nrg1, and many of the *nrg1Δ* mutant phenotypes were not as severe as mutations in the more upstream components of the cAMP pathway [37]. Therefore, we hypothesized that several transcriptional regulators would control capsule gene induction in response to the PKA pathway. Using a combination of bioinformatic and phenotypic screening, we identified the *C. neoformans* Rim101 protein as another potential novel PKA-dependent transcriptional regulator of capsule genes.

We hypothesized that the *C. neoformans* Rim101 protein may be a target of direct PKA phosphorylation due to the presence of a consensus sequence for PKA phosphorylation at amino acid positions 730–736. In contrast, the previously described *C. albicans* and *S. cerevisiae* Rim101 proteins do not contain potential PKA phosphorylation consensus sequences. However, there are multiple ways in which PKA can regulate downstream targets, including indirect activation of upstream regulatory proteins as well as by occupying the chromatin of the target genes [72]. Our bioinformatic approach, therefore, does not identify all of the targets of PKA, but does allow us to potentially identify direct targets of PKA phosphorylation.

To determine the relationship between Rim101 and PKA, we used complementary genetic, biochemical, and protein localization experiments. Our results suggest that PKA and Rim20 are necessary for maintenance of Rim101 nuclear localization by altering the cleavage of this transcription factor. Rim20 has been previously implicated in the first cleavage of Rim101, by binding to PEST domains, which are also present in *C. neoformans* Rim101 [43],[45]. In contrast to the predominantly nuclear localization of Rim101 in wild-type cells, we observed both nuclear and cytoplasmic localization of this protein in the *pka1* and *rim20* mutant strain backgrounds. We also observed both nuclear and cytoplasmic localization of the Rim101-S773A mutant protein with a putative PKA phosphorylation consensus sequence mutation. In addition, the GFP-tagged Rim101 protein in all of these strains had decreased electrophoretic mobility when compared to the *rim101Δ* *+Gfp-RIM101* strain. The larger band is not due to hyperphosphorylation as this mobility shift was not reversed by treatment with phosphatase. Together, these data indicate that both the cAMP/PKA pathway and the Rim pathway are involved in *C. neoformans* Rim101 processing and cellular localization.

In *Aspergillus* and *Candida*, PacC/Rim101 is activated by two cleavage events, first mediated Rim20 and Rim13 and second by the proteosome [43],[45],[53]. We demonstrate that *C. neoformans* Rim101 activation may also occur in response to two protein cleavage events, as the Rim101 protein is further cleaved from the 120kD form to a 70kD form in capsule inducing conditions. This further cleavage was not observed when *PKA1* or *RIM20* were disrupted, suggesting that the initial cleavage to 120 kD is necessary for further processing and activation of Rim101. The multiple smaller bands/laddering observed when *PKA1* or *RIM20* are disrupted may indicate altered proteosome-mediated processing events, suggesting that both Pka1 and Rim20 are necessary to cause appropriate proteosomal involvement and maintain the balance between processing and degradation. This is consistent with data from *A. nidulans*, where PacC is first converted by PalB and PalA under alkaline conditions to a 53kD intermediate which exposes the second processing site to the proteosome [53]. Hervas-Aguilar et al. also demonstrated that phosphorylation can accumulate on the 72 and 53kD PacC intermediates during alkaline conditions and affect processing. This is consistent with our results that PKA is involved in regulating processing of Rim101 in *C. neoformans*, although there is large divergence in the C-terminal and in the potential signaling motifs between these orthologous proteins in these distantly related species. Interestingly, capsule-inducing conditions are not alkaline and thus are not a traditional activating condition for Rim101 proteins. Therefore, CnRim101 may have acquired novel activating conditions in order to respond to the specific host conditions experienced by cryptococcal cells *in vivo*.

When we examined the targets of Rim101 transcriptional activation, we found that many Rim101 downstream targets and responses from other pathogenic fungi, such as *C. albicans*, are conserved in *C. neoformans*. We demonstrated that CnRim101 is important for growth under alkaline conditions *in vitro*. Using comparative transcriptional profiling, we determined that *ENA1*, a known downstream target of Rim101 in other fungal species, showed decreased expression in the *rim101Δ* mutant strain (Table 2). The promoter of the *ENA1* gene also had a conserved predicted Rim101 binding sequence, suggesting that it might be a direct target of Rim101 in *C. neoformans*, unlike in *S. cerevisiae,* where Rim101 regulates *ENA1* through Nrg1 [37]. Idnurm et al. showed that Ena1 is required for *C. neoformans* survival under alkaline conditions, and that appropriate response to alkaline conditions is necessary for virulence of *C. neoformans* [73]. Therefore, decreased expression of *ENA1* in the *rim101Δ* mutant strain may explain the defect in alkaline growth of the *rim101Δ* mutant.

Extracellular pH is involved in regulating iron uptake genes through the Rim101 pathway in *C. albicans* and *S. cerevisiae* [3], [5]–[7],[15],[23],[25]. The relationship between iron homeostasis and Rim101 is also conserved in *C. neoformans*. In order to determine the mechanism for the *rim101Δ* mutant strain sensitivity to low iron, we compared the transcriptional profile between wild-type and the *rim101Δ* *mutant* strain after incubation in capsule-inducing conditions. Our microarray analysis concluded that a number of iron homeostasis genes are differentially regulated between the *rim101Δ* mutant and the wild-type and we confirmed these alterations in gene expression using quantitative real-time PCR. When we examined the putative promoter regions of the candidate genes, we discovered potential Rim101 consensus binding sequences in *CFT1, FET3,* and *SIT1* among others, suggesting these genes are direct targets of Rim101. Similarly, in *C. albicans*, Rim101 binds directly to the promoter region of the ferric reductase genes *FRE1* and *FRP1* to cause increased transcription under iron-limited environments [23].

In *C. neoformans*, iron uptake is regulated by two pathways: PKA and Cir1. Transcriptional profiling showed that many iron genes, such as the iron permease Cft1 and reductase Cfo1 are differentially regulated by PKA [24],[26],[60],[61]. We have demonstrated that Rim101 is regulated by PKA, thus providing a mechanism for PKA regulation of these iron genes. However, in our transcriptional profiling, we did not demonstrate any difference in expression of Cir1 in the *rim101Δ* mutant strain, further suggesting that there are two pathways that regulate iron homeostasis. In *C. albicans*, two signaling pathways regulate iron homeostasis in response to different forms of iron limitation. In *C. albicans*, the ferric reductase gene *FRP1* is differentially regulated by Rim101 and by CBF transcription factors in response to different forms of iron limitation [23]. It is possible that *C. neoformans* has a similar set of transcription factors to regulate the expression of these iron homeostasis genes under different iron-limiting environments, and that the cell uses both Cir1 and Rim101 to regulate the expression of Cft1 under different environmental stimuli and iron source limitations.

Despite the decreased surface capsule observed in the *rim101Δ* mutant cells when stained with India ink, this strain was still able to secrete glucuronoxylomannan (GXM) at a similar size and concentration as wild type when the cells were grown in capsule inducing conditions. This data does not preclude other differences in structure and modifications to the GXM in the mutant strain. In accordance with the amount of secreted polysaccharide from the *rim101Δ* mutant strain, our transcriptional profiling revealed that few capsule biosynthesis genes are transcriptionally regulated by Rim101. In the *rim101Δ* mutant strain we observed decreased expression of UDP-glucose dehydrogenase Ugd1, mannosyltransferase Cmt1, and phosphomannomutase [55]–[58]. Unlike the iron uptake genes, these capsule biosynthesis genes do not have conserved Rim101 binding sites in the promoter regions, suggesting that these are not direct targets of Rim101. Therefore, our data indicates that CnRim101 is required for the transcriptional activation of some genes involved in capsule biosynthesis; however, the most important effects of Rim101 on capsule are likely due to changes in polysaccharide binding to the cell surface. We hypothesize that Rim101 regulates capsule by altering the expression of genes responsible for anchoring capsule to the cell wall, rather than acting as a direct regulator of these capsule biosynthesis genes.

Unexpectedly for an acapsular strain, the *rim101Δ* mutant displayed no attenuation in virulence in the mouse inhalation model of cryptococcosis. This confirms prior broad screening experiments of *C. neoformans* mutants to identify genes required for survival within mice [39]. In these studies, the *rim101Δ* strain was slightly more virulent than wild-type, as we demonstrated here. Follow-up experiments determining fungal load in the brain, lung, and spleen showed no defects in dissemination. When Rim101 is mutated in *Candida,* the resulting strains are avirulent as Rim101 regulates processes necessary for fungal virulence [3],[14],[19]. In a fungal pathogen of plants, *Fusarium oxysporum,* a *rim101Δ* mutant strain is more virulent than wild-type due to the derepression of acid response genes conferring a survival advantage in the acidic host environment of the tomato [2]. Similarly, our data indicates that the *C. neoformans rim101Δ* mutant grows better than wild-type within the acidic phagolysosome of the activated macrophage [29],[74]. Perhaps the derepression of acid responsive genes in the *rim101Δ* mutant could explain the increased growth within the acidic phagolysosome and thus within the lungs of the infected host. Another explanation for the retained virulence of the *rim101Δ* mutant strain is that the capsular polysaccharide may be shed into the surrounding tissues. This capsular material has well defined immunosuppressant effects. Capsular polysaccharide has even recently been used as an experimental therapy for autoimmune diseases such as rheumatoid arthritis [75]. Therefore, the retained virulence may be attributed to the profound immunomodulatory effects of strains that produce and secrete large amounts of capsule. Also, not all capsule-defective *C. neoformans* strains are hypovirulent in model systems. The acapsular *ags1Δ* mutant is fully virulent in the nematode model of cryptococcosis, although sensitive to temperature and thus avirulent in the mouse [49],[50]. The virulence of these strains suggests that capsule may be playing an important role in suppressing the immune system, even when not bound to the cell as an anti-phagocytic mechanism.

It is also possible that the hypocapsular *rim101Δ* mutant may present an altered cell surface for immune recognition, exposing different antigens resulting in a substantively different immune response than for an encapsulated WT strain. In this model, the increased virulence might result from alterations of the exposed *C. neoformans* surface antigens leading to over-stimulation of the immune system, such as seen in the response to β-glucan in the *C. albicans* cell wall [76],[77]. In our microarray data we observed increased expression of MP88 and MP98, two immuno-dominant mannoproteins, in the *rim101Δ* mutant strain, further supporting a model of an altered antigen surface on the fungus as a result of absent Rim101 activity [78],[79]. MP88 has also been documented as having increased expression in a *pka1Δ* mutant strain, which may be due to decreased Rim101 activity [60]. A more detailed evaluation of the nature of this cellular infiltration into the infected lungs will help define the varied immune response to different *C. neoformans* strains.

In summary, we have demonstrated that the *C. neoformans* Rim101 transcription factor retains conserved functions with orthologous proteins from other fungal species, such as regulation of pH response, cell wall formation, and iron homeostasis. However, the phenotypic output resulting from a *C. neoformans* Rim101 mutation supports the hypothesis that this conserved protein has been co-opted for unique, species-specific function. In contrast to other fungal species such as *Candida* or *Aspergillus* that have adapted to the neutral/alkaline pH of the host lungs and use Rim101 as an inducing signal for virulence, *C. neoformans* may be better adapted for acidic microenvironments in the host, such as the macrophage phagolysosome. Moreover, our experiments demonstrating PKA regulation of CnRim101 further suggests that conserved signaling elements can be regulated in novel ways to allow adaptation of microorganisms to specific niches in the environment of the infected host.

## Materials and Methods

### Strains and media

*Cryptococcus neoformans* strains used in this study are listed in Table 1. All *C. neoformans* strains were created in the H99 strain background unless otherwise stated. Strains were maintained on YPD (1% yeast extract, 2% peptone, 2% glucose), a standard yeast medium. Selective media contained nourseothricin (100 mg/L Werner BioAgents, Jena-Cospeda, Germany) or neomycin (G418) (200 mg/L Clontech, Takara-Bio Inc.). Capsule inducing medium (Dulbecco's modified Eagle's media with 25 mM NaHCO_3_) was prepared as previously described [31]. YNB media (0.67% yeast nitrogen base without amino acids, 2% glucose) was prepared as previously described [33]. Alkaline pH media were created by buffering YNB with 25 mM NaMOPS and adjusting to target pH with NaOH. Resistance to hydrogen peroxide was tested by disc diffusion as described previously [28]. Niger seed agar was prepared from 70 g Niger seed extract (Niger seed pulverized and boiled for 15 min and filtered through cheesecloth) and 4% Bacto agar as previously described [33].

### Molecular biology techniques

Standard techniques for Southern hybridization were performed as described [80]. *C. neoformans* genomic DNA for Southern blot analysis was prepared using CTAB phenol-chloroform extraction as described [81].

### Gene disruption

The wild-type *RIM101* gene (NCBI GeneID CNH0097) was mutated using biolistic transformation and homologous recombination with a *rim101::nat* mutant allele in which the entire *RIM101* coding region was precisely replaced with the nourseothrycin resistance gene (*nat*) [82],[83]. The *rim101::nat* mutant allele was created using PCR overlap extension as described [84]. Several *rim101Δ* mutants from independent transformation events displayed identical phenotypes *in vitro*; therefore one strain (TOC2) was chosen as the *rim101* strain for the presented experiments. Putative deletion strains were confirmed by PCR and Southern blot analysis. A second *rim101Δ* strain was made by creating an identical *rim101Δ* disruption construct first created by Liu et. al [39] and biolistic transforming it into the H99 strain background. This strain has a partial deletion of the *RIM101* gene. Putative mutant strains were confirmed by PCR and Southern blot analysis, and phenotypically compared to the full TOC2 deletion strain.

To reconstitute the wild-type allele, the *RIM101* locus was amplified from the H99 wild-type strain using primers (5′ CTGTATCCTTCACTTGAGGC 3′) and (5′ AGCTGTGCGTATCCAATAAT 3′). The neomycin resistance allele was also amplified separately using the M13 forward and reverse primers and both alleles were transformed using biolistic transformation into strain TOC2 to make the reconstituted strain TOC4 as described previously [85]. The reconstituted strain was tested by PCR for presence of the wild-type allele, and examined for reversion of the mutant phenotypes.

The wild-type *RIM20* allele (NCBI GeneID CNG00250) was similarly mutated with a *rim20::nat* mutant allele created by PCR overlap extension. Several *rim20Δ* mutants from independent transformation events displayed identical phenotypes *in vitro*; therefore one strain (TOC14) was chosen as the *rim20* strain for the presented experiments. This strain was confirmed using PCR and Southern analysis.

### Protein localization/GFP fusion

We created a green fluorescent protein (Gfp)-Rim101 fusion protein to examine the subcellular localization of Rim101. A histone H3 promoter-GFP fusion [86] was cloned into the neomycin-resistance containing plasmid pJAF, utilizing BamHI and EcoRI to make the resulting plasmid, pCN50. The coding region and the terminator sequence of *RIM101* was amplified using primers modified with BamHI sites (5′-AGTTAGGATCCATGGCTTACCCAATTCTCCC-3′ and 5′-ACTGATGGATCCGAGGAAAGCGTCAAGGATATG-3′). The *RIM101* gene was then cloned into the pCN50 plasmid at the BamHI site to create the plasmid pTO2 in which the Gfp-Rim101 fusion protein is constitutively expressed under the His3 promoter. pTO2 was then biolistically transformed into *C. neoformans* strain TOC2, JKH7, CDC7, and TOC17 as previously described to create strains TOC10, TOC12, TOC13 and TOC21 respectively. pTO2 was mutated into pTO3 using PCR-mediated site-directed mutagenesis to change serine 773 to alanine, using primers 5′-GAGAGTGATGCCGCACGTCGATACTGTCCTG-3′ and 5′- AGTTAAGATCTATGGCTTACCCAATTCTCCC-3′. pTO3 was then biolistically transformed into TOC2, CDC7 and TOC17 to create strains TOC18, TOC20 and TOC22 respectively.

### Microscopy

Bright field, differential interference microscopy (DIC) and fluorescent images were captured with a Zeiss Axio Imager.A1 fluorescent microscope equipped with an AxioCam mrM digital camera. Confocal images were captured using a Zeiss LSM inverted confocal microscope with the Argon/2 488 laser at×100 magnification. To visualize capsule, cells were grown in inducing conditions (described above), then stained with India ink on glass slides. Images were collected at ×63 magnification. To visualize GFP, cells were washed three times in PBS, and images were collected at ×63 magnification, using 488 nm wavelength for fluorescence. The strains exhibited significant artifactual fluorescence signal when fixed and DAPI stained, therefore all fluorescence images were taken without fixing, precluding DAPI staining.

### Capsule blotting

Estimation of shed capsule polysaccharide size and amount was performed using a technique described by Yoneda and Doering [47]. Conditioned media was made by growing strains in Dulbecco's modified Eagle's medium or low iron medium for 1 week at 30°C with shaking. The cells were incubated at 70°C for 15 minutes to denature enzymes, then pelleted for 3 minutes at 1500 rpm. The resulting supernatant was sterile filtered using a 0.2u filter. 15 uL of conditioned media was mixed with 6x loading dye and run on an agarose gel at 25V for 15 h. The polysaccharides were then transferred to a nylon membrane using Southern blotting techniques. The membrane was air-dried and blocked using Tris-Buffered Saline-Tween-20 (TBS-t) with 5% milk. To detect the polysaccharide, the membrane was incubated with monoclonal antibody 18b7 (1/1000 dilution) [87], washed with TBS-t, then incubated with an anti-mouse peroxidase-conjugated secondary antibody (1/25,000 dilution, Jackson Labs) and detected using SuperSignal West Fempto Maximum Sensitivity Substrate (ThermoScientific).

### Protein extraction, immunoprecipitation and western blot analysis

Protein extracts were obtained using a method previously described [88]. Briefly, cells were incubated to an optical density at 600 nm of 1 in YPD and capsule-inducing conditions. Twenty-milliliter samples of growing cells were pelleted and resuspended in 0.5 mL of lysis buffer containing 2x protease inhibitors (Complete, Mini, EDTA-free; Roche) and 2x phosphatase inhibitors (PhosStop; Roche). To lyse the cells, the supernatant was removed and the cells were lysed by bead beating (0.5 mL of 3uM glass beads in a Mini-BeadBeater-16 (BioSpec), 4 cycles for 30 s each). Following lysis, the samples were immunoprecipitated using 1.6µg anti-GFP antibody (Roche) for 1 hour, then rotated with 80µL protein G Sepharose (Thermo Scientific) for 1 hour. After washing, the samples were eluted by the addition of 40µL 5x Laemmli sample buffer and boiling. Western blots were performed as described previously, using NuPAGE Tris-Acetate gels or NuPAGE Bis-Tris gels to separate the samples. To detect the GFP-labeled proteins, the blots were incubated with anti-GFP primary antibody (1/5,000 dilution) and an anti-mouse peroxidase-conjugated secondary antibody (1/25,000 dilution, Jackson Labs). As a control for non-specific immunoprecipitation, samples were tested in a mock IP (in which no antibody was added) and probed with the anti-GFP antibody, and no bands were observed in this control experiment.

### RNA and cDNA preparation

Strains were incubated to mid-logarithmic phase in YPD then washed three times with sterile water before incubation in capsule-inducing media for 3 hours. Cells were washed three times before centrifugation and freezing on dry ice and lyophilizing. RNA was prepared from lyophilized samples using the RNeasy kit (Qiagen). cDNA for real time-PCR was generated using RETROscript (Ambion) using oligo-dT primer. Quantitative real-time PCR was performed as previously described, using the constitutive *GPD1* gene to normalize the samples [37].

### Microarray and data analysis

The microarray used in this study was developed by the Cryptococcus Community Microarray Consortium with financial support from individual researchers and the Burroughs Wellcome Fund (http://genome.wustl.edu/services/microarray/cryptococcus_neoformans). RNA labeling and hybridization were performed by the Duke University Microarray Core Facility according to their established protocols for custom spotted arrays [37],[89]. Data was analyzed using JMP genomics (SAS institute, Cary NC) and initial background subtraction was performed. We used ANOVA normalization and FDR analysis to calculate differences between treatment effects for pairs of inducing conditions. Genes were considered for further evaluation if they showed log_2_-transformed fold changes with a *p-*value <0.02.

### Virulence and data analysis

The virulence of the *C. neoformans* strains was assessed using a murine inhalation model of cryptococosis as described previously [70]. 10 female A/Jcr mice were inoculated intranasally with 5×10^5^ *C. neoformans* cells of wild-type (H99), *rim101Δ*mutant (TOC2), or *rim101+RIM101* complemented strain (TOC4). Mice were monitored daily for signs of infection and were sacrificed at predetermined clinical endpoints predicting mortality.

The statistical significance between the survival curves of all animals infected with each strain was evaluated using the log-rank test (JMP software, SAS institute, Cary NC). Cell counts were analyzed using Student's *t-*test. All studies were performed in compliance with the institutional guidelines for animal experimentation.

Survival within alveolar macrophage-like J744.1 cells was tested by aliquoting 50 ul of 1×10^5^ macrophage cells/mL into wells in a 96-well plate. The cells were activated by adding LPS and INFγ and incubating overnight. 50 ul of 2×10^6^cells/mL of each cryptococcal strain were added to the wells and co-incubated for 1 hour after which the excess cells were removed and fresh media was added. The engulfed cells were incubated for 24 hours then disrupted with 0.5% SDS for 5 minutes to lyse the macrophages. The media was removed, and the well was washed 2× with 100 uL PBS. The washes were combined and diluted at 1∶100 before plating. Plates were incubated for 2 days before colony counts [70].

## Supporting Information

Table S1 Complete data set of all genes differentially regulated in the *rim101* mutant strain
(0.88 MB DOC)
