# Supplementary material for: Interaction of Cryptococcus neoformans Rim101 and Protein Kinase A Regulates Capsule
Source: PLoS Pathog. 2010 Feb 19;6(2):e1000776. doi: 10.1371/journal.ppat.1000776 (PMC2824755; doi:10.1371/journal.ppat.1000776)
Supplement: Table S1 — Complete data set of all genes differentially regulated in the rim101 mutant strain (0.88 MB DOC) [file ppat.1000776.s001.doc]

| **LOCUS** | **ANNOTATION** | **DIFF OF TREATMENT** | **LOG_10_ P VALUE** | **Fold change (WT/rim101)** |
| --- | --- | --- | --- | --- |
| CNC01660 | cytokine inducing-glycoprotein, putative | 8.88 | 4.96 | 471.88 |
| CNI00030 | hypothetical protein unique to crypto | 6.47 | 3.65 | 88.73 |
| CNL05450 | sodium:inorganic phosphate symporter, putative | 6.35 | 3.58 | 81.68 |
| CNE04530 | siderochrome-iron transporter, putative | 6.34 | 4.94 | 81.14 |
| CNH03490 | expressed protein CIG | 6.24 | 5.98 | 75.61 |
| CND04470 | expressed protein | 6.19 | 4.48 | 73.15 |
| CNH03500 | hypothetical protein unique to crypto | 5.45 | 5.36 | 43.66 |
| CNB05770 | xenobiotic-transporting ATPase, putative | 4.38 | 4.54 | 20.77 |
| CNM02420 | acidic laccase, putative | 4.37 | 3.98 | 20.75 |
| CNM02430 | CFT1 | 4.05 | 4.97 | 16.59 |
| CNC03810 | hypothetical protein | 4.01 | 5.12 | 16.17 |
| CNG00950 | metalloreductase, putative | 4.01 | 4.54 | 16.09 |
| CNB00400 | STR3 | 3.95 | 4.48 | 15.45 |
| CNB02410 | expressed protein | 3.93 | 3.60 | 15.28 |
| CNA08170 | hypothetical protein | 3.62 | 3.93 | 12.33 |
| CNM00090 | glycoprotein, putative | 3.58 | 7.60 | 11.95 |
| CNA07920 | siderochrome-iron (ferrioxamine) uptake transporter, putative | 3.57 | 5.78 | 11.84 |
| CNH00970 | transcription factor PacC, putative | 3.50 | 5.68 | 11.30 |
| CNC06440 | inositol-3-phosphate synthase, putative | 3.49 | 4.47 | 11.22 |
| CNC00200 | conserved hypothetical protein | 3.40 | 5.04 | 10.54 |
| CNB03640 | oxidoreductase, putative | 3.23 | 5.10 | 9.40 |
| CNK01300 | hypothetical protein | 3.21 | 2.99 | 9.23 |
| CNB00400 | unknown | 3.18 | 4.61 | 9.05 |
| CNA04360 | hypothetical protein | 3.07 | 5.95 | 8.37 |
| CNA05220 | membrane transport protein, putative | 3.02 | 3.43 | 8.13 |
| CNH02130 | hypothetical protein | 3.01 | 2.13 | 8.07 |
| CNM01890 | unknown | 2.95 | 2.88 | 7.73 |
| CNF01190 | expressed protein | 2.95 | 2.48 | 7.71 |
| CNG00120 | hypothetical protein | 2.89 | 5.71 | 7.39 |
| CNK02580 | ribonucleoside-diphosphate reductase, putative | 2.87 | 3.24 | 7.33 |
| CNJ01880 | ammonium transporter, putative | 2.87 | 4.61 | 7.33 |
| CNK02970 | mitotic chromosome condensation-related protein, putative | 2.83 | 5.27 | 7.13 |
| CND01080 | copper uptake transporter, putative | 2.82 | 4.12 | 7.09 |
| CNK00170 | Tubulin gamma chain (Gamma tubulin), putative | 2.81 | 4.30 | 7.01 |
| CNA05130 | ENA1 | 2.79 | 4.77 | 6.91 |
| CNE02260 | expressed protein | 2.79 | 3.22 | 6.91 |
| CNI00360 | oxidoreductase, putative | 2.65 | 2.70 | 6.26 |
| CNC06900 | expressed protein | 2.64 | 3.49 | 6.24 |
| CNC00440 | conserved hypothetical protein | 2.63 | 5.54 | 6.21 |
| CNK02590 | mismatch repair-related protein, putative | 2.63 | 5.13 | 6.21 |
| CNI00900 | succinate-semialdehyde dehydrogenase (NAD(P)+), putative | 2.63 | 2.12 | 6.19 |
| CNA02400 | conserved hypothetical protein | 2.62 | 3.82 | 6.13 |
| CND03730 | unknown | 2.60 | 2.04 | 6.07 |
| CNN02350 | hypothetical protein | 2.60 | 2.57 | 6.07 |
| CNM02380 | expressed protein | 2.60 | 5.93 | 6.06 |
| CNA03450 | expressed protein | 2.57 | 2.76 | 5.95 |
| CNJ01230 | thymidylate synthase | 2.56 | 2.52 | 5.89 |
| CNJ00860 | conserved hypothetical protein | 2.54 | 4.91 | 5.80 |
| CNC06570 | unknown | 2.53 | 2.48 | 5.76 |
| CNA01720 | expressed protein | 2.52 | 4.12 | 5.75 |
| CNG02550 | hypothetical protein | 2.52 | 4.23 | 5.75 |
| CND06030 | unknown | 2.48 | 3.52 | 5.57 |
| CNB01920 | iron ion transporter, putative | 2.48 | 3.84 | 5.56 |
| CNA06580 | myosin-like protein nuf2, putative | 2.47 | 2.35 | 5.55 |
| CNI03050 | hypothetical protein | 2.46 | 5.38 | 5.49 |
| CND06030 | FRP | 2.46 | 3.45 | 5.49 |
| CNN02360 | hypothetical protein | 2.45 | 2.84 | 5.45 |
| CND00380 | glucosidase, putative | 2.41 | 3.71 | 5.33 |
| CND02310 | hypothetical protein | 2.37 | 2.85 | 5.18 |
| CNA04310 | 3-isopropylmalate dehydrogenase, putative | 2.37 | 3.56 | 5.17 |
| CNE01300 | conserved hypothetical protein | 2.36 | 4.80 | 5.13 |
| CNC00400 | cytoplasm protein, putative | 2.36 | 2.69 | 5.12 |
| CNG01880 | hypothetical protein | 2.35 | 5.83 | 5.10 |
| CNI02790 | vacuole protein, putative | 2.32 | 3.44 | 4.98 |
| CNL06530 | conserved hypothetical protein | 2.28 | 2.85 | 4.87 |
| CNH00420 | sister chromatid cohesion-related protein, putative | 2.28 | 3.10 | 4.87 |
| CNC07120 | protein regulator of cytokinesis 1, putative | 2.28 | 2.53 | 4.85 |
| CND03740 | aldo-keto reductase, putative | 2.28 | 3.88 | 4.85 |
| CND03600 | conserved hypothetical protein | 2.28 | 6.41 | 4.84 |
| CNE03460 | expressed protein | 2.27 | 3.94 | 4.84 |
| CNA04560 | hypothetical protein | 2.27 | 4.75 | 4.84 |
| CNA05000 | conserved hypothetical protein | 2.26 | 2.55 | 4.80 |
| CNF01800 | chitin deacetylase, putative | 2.26 | 4.24 | 4.78 |
| CNH00780 | unknown | 2.25 | 2.50 | 4.75 |
| CNF04360 | hypothetical protein | 2.24 | 2.91 | 4.72 |
| CNM00170 | asparaginase, putative | 2.23 | 3.69 | 4.71 |
| CNK00130 | thioredoxin peroxidase, putative | 2.23 | 2.87 | 4.70 |
| CNN01470 | hypothetical protein | 2.23 | 3.38 | 4.70 |
| CNG02090 | conserved hypothetical protein | 2.20 | 4.94 | 4.60 |
| CNG04000 | hypothetical protein | 2.20 | 3.51 | 4.60 |
| CNB03950 | conserved hypothetical protein | 2.19 | 2.91 | 4.58 |
| CNE02670 | conserved hypothetical protein | 2.18 | 3.81 | 4.53 |
| CND03300 | conserved hypothetical protein | 2.18 | 3.24 | 4.52 |
| CND04340 | vacuolar protein, putative | 2.16 | 2.58 | 4.46 |
| CNA03540 | expressed protein | 2.15 | 4.14 | 4.45 |
| CNE04470 | expressed protein | 2.15 | 4.51 | 4.45 |
| CND00530 | urea transporter, putative | 2.14 | 2.26 | 4.41 |
| CNM01420 | long-chain fatty acid transporter, putative | 2.14 | 5.48 | 4.39 |
| CNM01430 | dUTP diphosphatase, putative | 2.13 | 4.27 | 4.37 |
| CNF04110 | expressed protein | 2.13 | 4.19 | 4.37 |
| CNC04510 | STR3 | 2.12 | 4.76 | 4.33 |
| CNL03990 | stomatin-like protein, putative | 2.11 | 3.12 | 4.32 |
| CNB01560 | tubulin binding protein, putative | 2.11 | 3.13 | 4.31 |
| CNI03710 | thiamine biosynthetic bifunctional enzyme, putative | 2.10 | 4.28 | 4.30 |
| CNL04700 | expressed protein | 2.10 | 2.99 | 4.30 |
| CNA01380 | peptidase, putative | 2.09 | 3.88 | 4.26 |
| CNE00870 | endoplasmic reticulum protein, putative | 2.09 | 4.21 | 4.26 |
| CNE00690 | nucleus protein, putative | 2.08 | 4.04 | 4.23 |
| CNH00780 | unknown | 2.07 | 5.89 | 4.21 |
| CNN01270 | hypothetical protein | 2.07 | 3.10 | 4.19 |
| CNK02500 | protein serine/threonine kinase, putative | 2.05 | 2.67 | 4.13 |
| CNA07260 | succinate-CoA ligase (ADP-forming), putative | 2.04 | 4.41 | 4.10 |
| CNH02930 | EF-hand calcium-binding protein, Caltractin-cdc31 subfamily, putative | 2.02 | 2.65 | 4.05 |
| CND04510 | hypothetical protein | 2.01 | 3.81 | 4.02 |
| CNL04080 | histone h2a variant, putative | 2.00 | 3.63 | 3.99 |
| CNJ01650 | Extracellular elastinolytic metalloproteinase precursor, putative | 2.00 | 3.73 | 3.99 |
| CNB04090 | unknown | 1.99 | 2.68 | 3.98 |
| CNE03980 | DNA replication helicase dna2, putative | 1.99 | 2.03 | 3.97 |
| CNJ01130 | conserved hypothetical protein | 1.99 | 3.78 | 3.97 |
| CNA05840 | glycosyl hydrolase, putative | 1.98 | 4.35 | 3.95 |
| CNG01910 | hypothetical protein | 1.98 | 3.35 | 3.94 |
| CNK00170 | Tubulin gamma chain (Gamma tubulin), putative | 1.98 | 2.99 | 3.94 |
| CNI03540 | signal transducer, putative | 1.98 | 4.24 | 3.93 |
| CNM00440 | ADP-ribosylation-like factor, putative | 1.98 | 3.55 | 3.93 |
| CNB05360 | ATP dependent DNA helicase, putative | 1.96 | 2.02 | 3.90 |
| CNK03270 | expressed protein | 1.94 | 4.62 | 3.84 |
| CNG03110 | conserved hypothetical protein | 1.94 | 3.46 | 3.83 |
| CNC04710 | hypothetical protein | 1.94 | 4.03 | 3.83 |
| CNC01690 | expressed protein | 1.93 | 2.50 | 3.82 |
| CNB02000 | tubulin-folding cofactor B, putative | 1.93 | 2.90 | 3.80 |
| CNG02850 | serine/threonine protein kinase MST4, putative | 1.93 | 2.61 | 3.80 |
| CNK02100 | mitochondrion protein, putative | 1.92 | 3.36 | 3.79 |
| CNC00810 | gamma-tubulin complex component 3 (gcp-3), putative | 1.92 | 2.55 | 3.78 |
| CNA06260 | conserved hypothetical protein | 1.92 | 4.95 | 3.77 |
| CNG02510 | hypothetical protein | 1.91 | 2.72 | 3.76 |
| CNI03990 | hypothetical protein | 1.91 | 2.30 | 3.75 |
| CNA04540 | expressed protein | 1.90 | 2.42 | 3.72 |
| CNB02490 | conserved hypothetical protein | 1.89 | 2.59 | 3.72 |
| CNF02720 | actin lateral binding protein, putative | 1.89 | 3.48 | 3.71 |
| CNA06880 | uracil DNA N-glycosylase, putative | 1.87 | 2.57 | 3.64 |
| CNK02450 | hypothetical protein | 1.86 | 2.60 | 3.64 |
| CNL05940 | Maltose O-acetyltransferase | 1.86 | 3.49 | 3.63 |
| CNB03980 | hexose transport-related protein, putative | 1.85 | 2.03 | 3.60 |
| CNI00800 | peptidase, putative | 1.85 | 3.00 | 3.60 |
| CND04340 | vacuolar protein, putative | 1.85 | 2.56 | 3.60 |
| CNF02170 | endoplasmic reticulum protein, putative | 1.84 | 3.67 | 3.58 |
| CND02690 | damaged DNA binding protein, putative | 1.84 | 3.07 | 3.58 |
| CNB04020 | AE016780 membrane protein, putative, putative | 1.83 | 2.04 | 3.56 |
| CNA04090 | expressed protein | 1.82 | 2.95 | 3.54 |
| CNE04460 | hypothetical protein | 1.81 | 2.24 | 3.51 |
| CNH01910 | kinase regulator, putative | 1.80 | 3.62 | 3.49 |
| CNG04300 | conserved hypothetical protein | 1.80 | 2.09 | 3.48 |
| CNE01100 | long-chain-fatty-acid--CoA ligase, putative | 1.79 | 2.59 | 3.47 |
| CNF03470 | formate dehydrogenase, putative | 1.79 | 2.66 | 3.46 |
| CNC03000 | conserved hypothetical protein | 1.79 | 3.04 | 3.45 |
| CNF04130 | DNA polymerase alpha catalytic subunit, putative | 1.78 | 3.48 | 3.44 |
| CNH03300 | microtubule binding protein, putative | 1.78 | 4.24 | 3.44 |
| CNA01130 | conserved hypothetical protein | 1.78 | 3.33 | 3.44 |
| CNA08130 | 2-hydroxyacid dehydrogenase, putative | 1.77 | 2.77 | 3.42 |
| CNE03510 | myosin, light chain 2, 20 kDa, putative | 1.77 | 4.98 | 3.40 |
| CND05550 | hypothetical protein | 1.76 | 4.21 | 3.40 |
| CNA03370 | expressed protein | 1.76 | 2.66 | 3.39 |
| CNG02380 | DNA replication licensing factor cdc19 (cell division control protein 19), putative | 1.76 | 4.56 | 3.38 |
| CNE04400 | cyclin-dependent protein kinase regulator, putative | 1.75 | 3.78 | 3.36 |
| CNA02630 | conserved hypothetical protein | 1.75 | 4.28 | 3.36 |
| CNC02450 | water channel, putative | 1.75 | 5.05 | 3.36 |
| CNC01750 | Cdc2 cyclin-dependent kinase, putative | 1.74 | 3.31 | 3.33 |
| CNH02950 | cytoplasm protein, putative | 1.74 | 4.54 | 3.33 |
| CNF02160 | protein kinase activator, putative | 1.73 | 3.00 | 3.33 |
| CNG02140 | mitotic spindle assembly -related protein, putative | 1.73 | 2.87 | 3.33 |
| CNB05090 | CHL1 helicase, putative | 1.73 | 2.64 | 3.33 |
| CNE02570 | succinate:fumarate antiporter, putative | 1.73 | 3.05 | 3.32 |
| CNE03560 | dihydrokaempferol 4-reductase, putative | 1.72 | 3.73 | 3.29 |
| CNB02480 | GPI anchor biosynthesis-related protein, putative | 1.72 | 2.97 | 3.29 |
| CNF01200 | heme oxygenase 2, putative | 1.72 | 3.42 | 3.29 |
| CNC06570 | unknown | 1.72 | 2.58 | 3.28 |
| CNH01950 | hypothetical protein | 1.72 | 3.35 | 3.28 |
| CNN02110 | chitin synthase, putative | 1.71 | 2.08 | 3.27 |
| CNE03350 | expressed protein | 1.71 | 2.14 | 3.26 |
| CNE03260 | transcriptional activator, putative | 1.70 | 3.69 | 3.26 |
| CNF02030 | conserved hypothetical protein | 1.70 | 2.60 | 3.26 |
| CNK01000 | conserved hypothetical protein | 1.70 | 2.82 | 3.25 |
| CNE04360 | fatty-acid synthase complex protein, putative | 1.70 | 4.48 | 3.24 |
| CNG03350 | hypothetical protein | 1.69 | 2.37 | 3.23 |
| CNM01060 | hypothetical protein | 1.69 | 4.49 | 3.23 |
| CNI01600 | pre-mRNA splicing factor, putative | 1.69 | 3.59 | 3.22 |
| CNK03430 | nucleus protein, putative | 1.69 | 4.58 | 3.22 |
| CNH01660 | small monomeric GTPase, putative | 1.68 | 2.61 | 3.20 |
| CNF02980 | mitochondrion protein, putative | 1.68 | 2.92 | 3.19 |
| CNH00170 | phosphomannomutase, putative | 1.67 | 3.97 | 3.19 |
| CNA01010 | expressed protein | 1.67 | 2.11 | 3.19 |
| CNE01170 | cation-transporting ATPase, putative | 1.67 | 3.46 | 3.18 |
| CNA00890 | palmitoyl-protein thioesterase, putative | 1.66 | 3.99 | 3.17 |
| CND02270 | conserved hypothetical protein | 1.66 | 2.34 | 3.17 |
| CNB04790 | conserved hypothetical protein | 1.66 | 2.41 | 3.16 |
| CNM02100 | expressed protein | 1.66 | 3.10 | 3.16 |
| CNK02190 | conserved hypothetical protein | 1.66 | 3.41 | 3.15 |
| CND03520 | bud site selection-related protein, putative | 1.65 | 3.05 | 3.14 |
| CNE04600 | leukotriene-A4 hydrolase, putative | 1.65 | 2.29 | 3.14 |
| CNF03620 | Beta-hexosaminidase precursor, putative | 1.65 | 2.60 | 3.14 |
| CND05520 | transcription factor, putative | 1.65 | 3.47 | 3.14 |
| CNI01590 | manganese superoxide dismutase, putative | 1.65 | 2.82 | 3.14 |
| CNA00900 | ATP dependent DNA helicase, putative | 1.65 | 3.28 | 3.13 |
| CNF00340 | conserved hypothetical protein | 1.65 | 2.75 | 3.13 |
| CNH01990 | expressed protein | 1.64 | 3.06 | 3.12 |
| CNC00620 | hypothetical protein | 1.64 | 2.93 | 3.12 |
| CNB01020 | mandelate racemase/muconate lactonizing enzyme, putative | 1.64 | 2.21 | 3.11 |
| CNN00130 | hypothetical protein | 1.64 | 4.02 | 3.11 |
| CNH01390 | hypothetical protein | 1.64 | 4.12 | 3.11 |
| CNA08160 | mitochondrion protein, putative | 1.63 | 2.99 | 3.10 |
| CNB05610 | expressed protein | 1.63 | 3.39 | 3.09 |
| CNA00430 | conserved hypothetical protein | 1.63 | 4.94 | 3.09 |
| CNI00020 | expressed protein | 1.62 | 2.66 | 3.08 |
| CNK02610 | hypothetical protein | 1.62 | 4.59 | 3.08 |
| CND04360 | conserved hypothetical protein | 1.62 | 4.20 | 3.08 |
| CNE02240 | expressed protein | 1.62 | 3.76 | 3.07 |
| CNL06120 | expressed protein | 1.62 | 2.86 | 3.07 |
| CNI00240 | enoyl-CoA hydratase, putative | 1.62 | 2.85 | 3.07 |
| CND04260 | hypothetical protein | 1.61 | 2.06 | 3.06 |
| CND03830 | conserved hypothetical protein | 1.61 | 3.34 | 3.06 |
| CNC02500 | ATPase, putative | 1.61 | 2.03 | 3.06 |
| CNG04180 | nuclear condensin complex protein, putative | 1.61 | 3.67 | 3.05 |
| CNE04140 | methionyl-tRNA formyltransferase, putative | 1.60 | 3.02 | 3.04 |
| CNB02120 | expressed protein | 1.60 | 3.80 | 3.04 |
| CNL06670 | Tubulin beta chain, putative | 1.60 | 4.33 | 3.03 |
| CNE01650 | conserved hypothetical protein | 1.60 | 2.45 | 3.02 |
| CNA06990 | recombinase, putative | 1.59 | 3.97 | 3.01 |
| CNH03550 | hypothetical protein | 1.59 | 2.55 | 3.01 |
| CNA01640 | glutamate carboxypeptidase protein, putative | 1.59 | 3.05 | 3.00 |
| CNB04180 | conserved hypothetical protein | 1.59 | 3.99 | 3.00 |
| CNA00750 | histone binding protein, putative | 1.58 | 4.82 | 2.99 |
| CNE01490 | cell division control protein 45, putative | 1.58 | 3.14 | 2.98 |
| CNH02910 | malate synthase, putative | 1.57 | 3.55 | 2.96 |
| CNA06180 | GTPase, putative | 1.57 | 3.25 | 2.96 |
| CNB00780 | DNA polymerase Delta, small subunit, putative | 1.56 | 2.85 | 2.95 |
| CNG02520 | chitin synthase 1, putative | 1.56 | 2.53 | 2.94 |
| CNC04470 | delta24(24-1) sterol reductase, putative | 1.55 | 3.20 | 2.93 |
| CNB04440 | DNA clamp loader, putative | 1.55 | 3.20 | 2.93 |
| CNN01600 | hsk1-interacting molecule 1, putative | 1.55 | 3.33 | 2.93 |
| CNH01280 | DNA-(apurinic or apyrimidinic site) lyase, putative | 1.55 | 3.98 | 2.92 |
| CNK00320 | hypothetical protein | 1.55 | 2.28 | 2.92 |
| CND04350 | protein transporter, putative | 1.55 | 2.79 | 2.92 |
| CNK01170 | DNA repair protein rad8, putative | 1.54 | 3.21 | 2.92 |
| CNF03530 | glycogen phosphorylase, putative | 1.54 | 2.90 | 2.91 |
| CNF00170 | expressed protein | 1.54 | 2.27 | 2.91 |
| CNE00820 | glycerol dehydrogenase, putative | 1.54 | 2.90 | 2.91 |
| CNC02130 | conserved hypothetical protein | 1.54 | 2.92 | 2.91 |
| CNK00590 | fork head homolog XFD-2, putative | 1.54 | 3.12 | 2.90 |
| CNM01340 | cytoplasm protein, putative | 1.53 | 3.51 | 2.89 |
| CNE01970 | conserved hypothetical protein | 1.53 | 3.43 | 2.89 |
| CNA08290 | C-8 sterol isomerase, putative | 1.53 | 3.34 | 2.89 |
| CNL06460 | UDP-glucose dehyrodgenase UGD1 | 1.52 | 4.20 | 2.87 |
| CNE01180 | conserved hypothetical protein | 1.51 | 3.59 | 2.85 |
| CNC02330 | expressed protein | 1.51 | 2.95 | 2.85 |
| CNN01570 | AMP binding protein, putative | 1.51 | 3.40 | 2.84 |
| CNB01250 | short-chain dehydrogenase, putative | 1.50 | 3.18 | 2.83 |
| CNE03500 | vesicle-mediated transport-related protein, putative YIP1 | 1.50 | 5.51 | 2.83 |
| CNL05190 | SWI/SNF related, matrix associated, actin dependent regulator of chromatin, subfamily a, member 3, putative | 1.50 | 2.88 | 2.83 |
| CNF02300 | Bromodomain and PHD finger-containing protein 3, putative | 1.49 | 2.01 | 2.81 |
| CNE03160 | expressed protein | 1.49 | 2.17 | 2.80 |
| CNC02650 | cytoplasm protein, putative | 1.49 | 2.55 | 2.80 |
| CNE02820 | unknown | 1.48 | 2.35 | 2.79 |
| CNN02390 | expressed protein | 1.48 | 2.23 | 2.79 |
| CNE05330 | conserved hypothetical protein | 1.48 | 3.16 | 2.79 |
| CNG03960 | MYT1 kinase, putative | 1.48 | 3.04 | 2.79 |
| CNC01700 | fumarate hydratase, putative | 1.48 | 5.04 | 2.79 |
| CNB00560 | histone h2b, putative | 1.47 | 3.14 | 2.78 |
| CNG03030 | S-adenosylmethionine transporter, putative | 1.47 | 3.78 | 2.77 |
| CNE04830 | conserved hypothetical protein | 1.47 | 3.14 | 2.77 |
| CNA05810 | nicotinamide mononucleotide permease, putative | 1.47 | 2.05 | 2.76 |
| CNE04190 | expressed protein | 1.45 | 2.27 | 2.74 |
| CNB00550 | histone H2A-1, putative | 1.45 | 2.74 | 2.74 |
| CNJ02470 | poly(A)-specific ribonuclease, putative | 1.45 | 3.62 | 2.73 |
| CNF02180 | acetyl-CoA carboxylase, putative | 1.45 | 4.08 | 2.73 |
| CNI02520 | conserved hypothetical protein | 1.45 | 2.61 | 2.73 |
| CNN00140 | hydrolase, putative | 1.45 | 2.66 | 2.72 |
| CNB01730 | oxoglutarate dehydrogenase (succinyl-transferring), putative | 1.44 | 2.88 | 2.72 |
| CNN01480 | hypothetical protein | 1.44 | 3.85 | 2.71 |
| CND02280 | oxidoreductase, putative | 1.44 | 3.69 | 2.71 |
| CNH02070 | protein kinase SNF, putative | 1.44 | 2.31 | 2.70 |
| CNI03330 | O-methyltransferase, putative | 1.43 | 2.05 | 2.70 |
| CNG02080 | succinyl-coa ligase alpha-chain, mitochondrial precursor, putative | 1.43 | 3.75 | 2.70 |
| CNI03330 | O-methyltransferase, putative | 1.43 | 4.23 | 2.70 |
| CNH00400 | B2-aldehyde-forming enzyme, putative | 1.43 | 2.29 | 2.69 |
| CNJ02870 | unknown | 1.41 | 4.22 | 2.66 |
| CNJ01730 | hypothetical protein | 1.41 | 4.05 | 2.66 |
| CNA06590 | mitotic chromosome condensation-related protein, putative | 1.41 | 2.01 | 2.66 |
| CNI02780 | hypothetical protein | 1.41 | 2.21 | 2.66 |
| CNI03210 | hypothetical protein | 1.41 | 2.04 | 2.66 |
| CNB02810 | unknown | 1.41 | 3.52 | 2.66 |
| CNF03170 | expressed protein | 1.40 | 2.86 | 2.65 |
| CNI03450 | expressed protein | 1.40 | 3.56 | 2.64 |
| CNI02230 | nad-dependent malic enzyme, putative | 1.40 | 2.52 | 2.64 |
| CNF02300 | Bromodomain and PHD finger-containing protein 3, putative | 1.40 | 4.19 | 2.63 |
| CNM01670 | conserved hypothetical protein | 1.39 | 2.64 | 2.63 |
| CND01550 | hypothetical protein | 1.39 | 4.15 | 2.62 |
| CNI03780 | expressed protein | 1.38 | 2.70 | 2.61 |
| CNJ00870 | hypothetical protein | 1.38 | 2.33 | 2.61 |
| CNI03070 | conserved hypothetical protein | 1.38 | 2.09 | 2.61 |
| CND05280 | hypothetical protein | 1.38 | 2.33 | 2.60 |
| CNB01040 | hypothetical protein | 1.38 | 2.58 | 2.60 |
| CNF03140 | retrotransposon nucleocapsid protein, putative | 1.38 | 2.75 | 2.60 |
| CND01820 | hypothetical protein | 1.38 | 5.11 | 2.60 |
| CND02500 | expressed protein | 1.38 | 2.37 | 2.60 |
| CNH02670 | expressed protein | 1.37 | 2.12 | 2.59 |
| CNC04630 | thymidylate kinase, putative | 1.37 | 2.87 | 2.59 |
| CNF04440 | hypothetical protein | 1.37 | 2.14 | 2.58 |
| CNH03650 | hypothetical protein | 1.36 | 2.37 | 2.57 |
| CNJ00710 | hypothetical protein | 1.36 | 4.06 | 2.57 |
| CNC05550 | hypothetical protein | 1.36 | 3.37 | 2.57 |
| CNM00950 | Aspartate aminotransferase, mitochondrial precursor, putative | 1.36 | 4.67 | 2.56 |
| CNJ00600 | response to drug-related protein, putative | 1.36 | 3.36 | 2.56 |
| CNH03200 | unknown | 1.35 | 2.51 | 2.56 |
| CNH00510 | CDP-diacylglycerol-inositol 3-phosphatidyltransferase, putative | 1.35 | 4.88 | 2.55 |
| CNA00420 | expressed protein | 1.35 | 2.68 | 2.55 |
| CNF01590 | unknown | 1.35 | 2.14 | 2.55 |
| CNF02260 | kinesin, putative | 1.35 | 2.16 | 2.54 |
| CNF03860 | mitochondrion protein, putative | 1.35 | 4.18 | 2.54 |
| CNG02230 | expressed protein | 1.35 | 2.06 | 2.54 |
| CND04450 | hypothetical protein | 1.34 | 3.02 | 2.53 |
| CNC00410 | hypothetical protein | 1.34 | 2.17 | 2.53 |
| CNK00560 | delta DNA polymerase, putative | 1.34 | 2.26 | 2.53 |
| CNI01610 | conserved hypothetical protein | 1.34 | 5.35 | 2.53 |
| CNK01440 | expressed protein | 1.33 | 3.87 | 2.52 |
| CNA00410 | expressed protein | 1.32 | 4.92 | 2.50 |
| CNM00350 | conserved hypothetical protein | 1.32 | 3.14 | 2.50 |
| CNI02060 | mandelate racemase/muconate lactonizing enzyme, putative | 1.32 | 2.93 | 2.49 |
| CNC05740 | Cell division control protein 23, putative | 1.32 | 2.69 | 2.49 |
| CNH02980 | chromatin binding protein, putative | 1.32 | 3.23 | 2.49 |
| CNJ00970 | hypothetical protein | 1.31 | 3.15 | 2.49 |
| CNB00570 | histone H3, putative | 1.31 | 2.99 | 2.48 |
| CNA00540 | hypothetical protein | 1.31 | 2.20 | 2.48 |
| CNB02640 | cohesin complex subunit psm1, putative | 1.30 | 2.81 | 2.47 |
| CNG02500 | PHD-finger protein, putative | 1.30 | 2.22 | 2.46 |
| CNC00300 | hypothetical protein | 1.30 | 2.06 | 2.46 |
| CND03760 | conserved hypothetical protein | 1.29 | 3.13 | 2.45 |
| CNA03150 | hypothetical protein | 1.29 | 2.25 | 2.45 |
| CND06130 | conserved hypothetical protein | 1.29 | 2.65 | 2.44 |
| CND00940 | conserved hypothetical protein | 1.29 | 3.10 | 2.44 |
| CND02690 | damaged DNA binding protein, putative | 1.29 | 2.49 | 2.44 |
| CNF01940 | expressed protein | 1.29 | 4.07 | 2.44 |
| CND04900 | conserved hypothetical protein | 1.28 | 3.66 | 2.44 |
| CNE01230 | copper chaperone, putative | 1.28 | 4.64 | 2.43 |
| CNA00130 | expressed protein | 1.28 | 2.93 | 2.43 |
| CNB04910 | leucyl aminopeptidase, putative | 1.28 | 2.41 | 2.42 |
| CNB05650 | dolichol kinase, putative | 1.28 | 4.07 | 2.42 |
| CNL06720 | expressed protein | 1.27 | 2.46 | 2.41 |
| CNJ02870 | unknown | 1.27 | 2.44 | 2.41 |
| CNN00190 | conserved hypothetical protein | 1.27 | 2.68 | 2.41 |
| CNE00400 | expressed protein | 1.27 | 4.35 | 2.41 |
| CNA01330 | actin ii (centractin-like protein), putative | 1.27 | 2.55 | 2.41 |
| CNA08350 | conserved hypothetical protein | 1.27 | 3.02 | 2.41 |
| CNC00130 | hypothetical protein | 1.27 | 2.15 | 2.41 |
| CNG01050 | hypothetical protein | 1.27 | 2.93 | 2.41 |
| CNC01990 | unknown | 1.27 | 3.91 | 2.40 |
| CNB04430 | L-methionine porter, putative | 1.26 | 3.42 | 2.40 |
| CND03770 | hypothetical protein | 1.26 | 5.20 | 2.40 |
| CNB05590 | hypothetical protein | 1.26 | 3.19 | 2.40 |
| CNF01320 | hypothetical protein | 1.26 | 3.07 | 2.40 |
| CNF00900 | hypothetical protein | 1.26 | 3.25 | 2.40 |
| CNG04650 | cytoplasm protein, putative | 1.26 | 2.61 | 2.39 |
| CND03080 | conserved hypothetical protein | 1.26 | 2.65 | 2.39 |
| CNC05760 | carboxypeptidase D, putative | 1.26 | 2.63 | 2.39 |
| CNG03710 | conserved hypothetical protein | 1.26 | 2.84 | 2.39 |
| CNG02600 | hypothetical protein | 1.25 | 2.01 | 2.37 |
| CND04050 | conserved hypothetical protein | 1.24 | 3.57 | 2.36 |
| CNF04720 | pim1 protein (poly(a)+ RNA transport protein 2), putative | 1.24 | 2.16 | 2.36 |
| CNL06070 | conserved hypothetical protein | 1.24 | 2.05 | 2.36 |
| CND04440 | hypothetical protein | 1.24 | 2.50 | 2.35 |
| CNL05050 | unknown | 1.23 | 2.70 | 2.35 |
| CNC05700 | high-affinity iron permease CaFTR1, putative | 1.23 | 3.65 | 2.35 |
| CNI04250 | expressed protein | 1.23 | 2.45 | 2.34 |
| CNE02370 | FK506 binding protein 2, putative | 1.23 | 4.19 | 2.34 |
| CNH02220 | expressed protein | 1.23 | 3.24 | 2.34 |
| CNC01990 | unknown | 1.22 | 2.89 | 2.34 |
| CND05980 | nucleoside transporter | 1.22 | 2.16 | 2.34 |
| CNF04730 | nonhistone protein 6, putative | 1.22 | 2.33 | 2.34 |
| CND04950 | hypothetical protein | 1.22 | 2.37 | 2.33 |
| CNM00620 | galactose-1-phosphate uridylyltransferase | 1.22 | 3.35 | 2.33 |
| CND06050 | hypothetical protein | 1.22 | 2.42 | 2.33 |
| CNF00500 | septin ring protein, putative | 1.22 | 2.45 | 2.32 |
| CNC04770 | endo-1,3(4)-beta-glucanase, putative | 1.22 | 2.50 | 2.32 |
| CNH01920 | expressed protein | 1.22 | 2.94 | 2.32 |
| CNA03250 | hst3 protein, putative | 1.22 | 4.12 | 2.32 |
| CNE04320 | phosphatidylinositol transporter, putative | 1.21 | 4.37 | 2.32 |
| CNJ02720 | expressed protein | 1.21 | 2.29 | 2.31 |
| CNG03100 | L-malate dehydrogenase, putative | 1.21 | 4.61 | 2.31 |
| CNB05490 | conserved hypothetical protein | 1.21 | 2.12 | 2.31 |
| CNF02490 | hypothetical protein | 1.21 | 2.85 | 2.31 |
| CNA03300 | hypothetical protein | 1.21 | 4.64 | 2.31 |
| CND06020 | unknown | 1.20 | 2.44 | 2.30 |
| CNM00880 | DNA polymerase processivity factor, putative | 1.20 | 2.95 | 2.30 |
| CNM00010 | transporter, putative | 1.20 | 3.57 | 2.30 |
| CNC04980 | capsular associated protein | 1.20 | 4.09 | 2.30 |
| CND05460 | hypothetical protein | 1.20 | 2.12 | 2.29 |
| CNG01770 | unknown | 1.20 | 3.84 | 2.29 |
| CND00530 | urea transporter, putative | 1.20 | 2.01 | 2.29 |
| CNG02470 | Activator 1 40 kDa subunit, putative | 1.19 | 2.84 | 2.28 |
| CNE02050 | expressed protein | 1.19 | 2.09 | 2.28 |
| CNH02150 | conserved hypothetical protein | 1.19 | 2.68 | 2.28 |
| CNA06340 | conserved hypothetical protein | 1.19 | 3.71 | 2.28 |
| CNB05390 | expressed protein | 1.18 | 2.81 | 2.27 |
| CND04110 | hypothetical protein | 1.18 | 4.70 | 2.27 |
| CNI02410 | unknown | 1.18 | 2.27 | 2.27 |
| CNE03430 | septin, putative | 1.18 | 2.80 | 2.26 |
| CNE04380 | hypothetical protein | 1.18 | 3.15 | 2.26 |
| CNI03690 | 2,4-dienoyl-CoA reductase (NADPH), putative | 1.18 | 2.28 | 2.26 |
| CNA06530 | carnitine/acyl carnitine carrier, putative | 1.17 | 2.51 | 2.26 |
| CNK02960 | conserved hypothetical protein | 1.17 | 2.84 | 2.26 |
| CNA01260 | expressed protein | 1.17 | 2.81 | 2.25 |
| CNC02640 | unknown | 1.17 | 2.26 | 2.24 |
| CNB04350 | cytoplasm protein, putative | 1.16 | 2.12 | 2.24 |
| CNF04190 | vesicular-fusion protein sec18, putative | 1.16 | 2.72 | 2.24 |
| CNL03780 | chaperone regulator, putative | 1.16 | 2.10 | 2.24 |
| CNE04200 | conserved hypothetical protein | 1.16 | 3.97 | 2.24 |
| CNC03950 | expressed protein | 1.15 | 2.71 | 2.22 |
| CND03960 | DNA repair-related protein, putative | 1.15 | 2.12 | 2.22 |
| CND00090 | ATP-binding cassette (ABC) transporter, putative | 1.15 | 3.48 | 2.21 |
| CNA06660 | hypothetical protein | 1.14 | 2.38 | 2.21 |
| CNJ02210 | spermine transporter, putative | 1.14 | 2.80 | 2.21 |
| CNB04890 | cytoplasm protein, putative | 1.14 | 2.33 | 2.21 |
| CNA00420 | expressed protein | 1.14 | 2.14 | 2.20 |
| CNH00650 | conserved hypothetical protein | 1.13 | 2.87 | 2.19 |
| CNB03860 | phosphoadenylyl-sulfate reductase (thioredoxin), putative | 1.13 | 2.53 | 2.19 |
| CND02670 | endoplasmic reticulum protein, putative | 1.13 | 2.11 | 2.19 |
| CNC04160 | pria protein precursor, putative | 1.12 | 3.83 | 2.18 |
| CNG03850 | protein kinase, putative | 1.12 | 2.42 | 2.18 |
| CNA00310 | expressed protein | 1.12 | 2.80 | 2.17 |
| CNB04450 | 4-nitrophenylphosphatase, putative | 1.12 | 2.00 | 2.17 |
| CNB03650 | UTP-hexose-1-phosphate uridylyltransferase, putative | 1.12 | 2.48 | 2.17 |
| CNH02100 | hypothetical protein | 1.12 | 2.60 | 2.17 |
| CND00430 | hypothetical protein | 1.11 | 3.11 | 2.16 |
| CNE03730 | cytoplasm protein, putative | 1.11 | 4.17 | 2.16 |
| CNJ01210 | mitochondrion protein, putative | 1.11 | 3.15 | 2.16 |
| CNG01780 | conserved hypothetical protein | 1.11 | 3.49 | 2.15 |
| CNC06330 | hypothetical protein | 1.11 | 3.06 | 2.15 |
| CNG01700 | hypothetical protein | 1.10 | 2.10 | 2.14 |
| CNG00030 | unknown | 1.10 | 2.73 | 2.14 |
| CNF03830 | epsilon DNA polymerase, putative | 1.10 | 2.34 | 2.14 |
| CNC01070 | glycine dehydrogenase mitochondrial precursor, putative | 1.09 | 3.59 | 2.13 |
| CND02680 | hypothetical protein | 1.09 | 2.80 | 2.13 |
| CNH02350 | protein-histidine kinase, putative | 1.09 | 3.24 | 2.13 |
| CNA06400 | chromatin remodeling-related protein, putative | 1.09 | 2.04 | 2.13 |
| CNM00940 | hypothetical protein | 1.09 | 2.40 | 2.13 |
| CNE00850 | flavin-containing monooxygenase, putative | 1.09 | 3.85 | 2.13 |
| CNB03450 | unknown | 1.09 | 2.78 | 2.13 |
| CND03130 | peroxin19 Pex19p, putative | 1.08 | 2.21 | 2.12 |
| CNB03340 | nonselective cation channel, putative | 1.08 | 3.00 | 2.12 |
| CNA05070 | conserved hypothetical protein | 1.08 | 3.90 | 2.12 |
| CNB03440 | conserved hypothetical protein | 1.08 | 2.02 | 2.12 |
| CNI03650 | protein phosphatase, putative | 1.08 | 2.10 | 2.11 |
| CNC02690 | mitochondrial inner membrane protein, putative | 1.08 | 2.28 | 2.11 |
| CNL04500 | hypothetical protein | 1.08 | 4.31 | 2.11 |
| CNF04630 | expressed protein | 1.08 | 2.32 | 2.11 |
| CNJ00400 | DNA topoisomerase type I, putative | 1.08 | 2.13 | 2.11 |
| CNA04890 | hypothetical protein | 1.08 | 2.42 | 2.11 |
| CNG01100 | thioredoxin-dependent peroxide reductase, putative | 1.08 | 3.74 | 2.11 |
| CNA00510 | citrate synthase, putative | 1.08 | 2.30 | 2.11 |
| CNN00190 | conserved hypothetical protein | 1.07 | 3.22 | 2.11 |
| CNE04580 | cytoplasm protein, putative | 1.07 | 2.18 | 2.10 |
| CNA01710 | retrograde transport, endosome to Golgi-related protein, putative | 1.07 | 2.33 | 2.10 |
| CNM01740 | hypothetical protein | 1.07 | 2.59 | 2.10 |
| CND05620 | hypothetical protein | 1.06 | 2.66 | 2.09 |
| CNH02240 | conserved hypothetical protein | 1.06 | 2.08 | 2.09 |
| CNC03320 | hypothetical protein | 1.06 | 2.03 | 2.09 |
| CNJ00730 | endopeptidase, putative | 1.06 | 2.32 | 2.09 |
| CNH02550 | conserved hypothetical protein | 1.06 | 4.13 | 2.09 |
| CNH02800 | protein tyrosine/threonine phosphatase, putative | 1.06 | 4.02 | 2.08 |
| CNA06220 | conserved hypothetical protein | 1.05 | 2.84 | 2.08 |
| CNH00880 | hypothetical protein | 1.05 | 2.14 | 2.07 |
| CNM02410 | conserved hypothetical protein | 1.05 | 2.92 | 2.07 |
| CND04330 | expressed protein | 1.05 | 2.27 | 2.07 |
| CNH03220 | conserved hypothetical protein | 1.04 | 2.37 | 2.06 |
| CNG04680 | hypothetical protein | 1.04 | 3.46 | 2.06 |
| CNF04200 | hypothetical protein | 1.04 | 3.08 | 2.06 |
| CNI00680 | hypothetical protein | 1.04 | 4.49 | 2.05 |
| CNK00980 | expressed protein | 1.04 | 2.20 | 2.05 |
| CNA07250 | unknown | 1.04 | 2.54 | 2.05 |
| CNB00690 | conserved hypothetical protein | 1.03 | 3.57 | 2.05 |
| CNB04350 | cytoplasm protein, putative | 1.03 | 2.30 | 2.05 |
| CNB04660 | hypothetical protein | 1.02 | 2.25 | 2.03 |
| CNI01200 | conserved hypothetical protein | 1.02 | 2.10 | 2.03 |
| CND03290 | hypothetical protein | 1.02 | 3.24 | 2.03 |
| CNC00190 | hydrolase, putative | 1.02 | 3.06 | 2.02 |
| CNG00420 | expressed protein | 1.02 | 4.37 | 2.02 |
| CNG03490 | malate dehydrogenase, putative | 1.02 | 4.71 | 2.02 |
| CNJ00900 | enoyl reductase, putative | 1.01 | 3.38 | 2.02 |
| CNC06000 | actin cross-linking, putative | 1.01 | 2.84 | 2.02 |
| CNI03710 | thiamine biosynthetic bifunctional enzyme, putative | 1.01 | 2.49 | 2.02 |
| CNA07250 | unknown | 1.01 | 3.06 | 2.01 |
| CNL03790 | expressed protein | 1.01 | 3.29 | 2.01 |
| CNH03270 | hypothetical protein | 1.01 | 3.10 | 2.01 |
| CNF02530 | peroxisomal hydratase-dehydrogenase-epimerase (hde), putative | 1.01 | 3.16 | 2.01 |
| CNL04910 | conserved hypothetical protein | 1.01 | 2.29 | 2.01 |
| CNM02390 | hypothetical protein | 1.00 | 3.20 | 2.01 |
| CNF04690 | endoplasmic reticulum protein, putative | 1.00 | 2.54 | 2.00 |
| CNM00050 | amino acid transporter, putative | 1.00 | 2.23 | 2.00 |
| CNC05590 | unknown | 1.00 | 3.72 | 2.00 |
| CNB01380 | damaged DNA binding protein, putative | 1.00 | 2.04 | 2.00 |
| CNM02470 | hypothetical protein | -1.00 | 2.50 | 2.00 |
| CNC02440 | cytokinesis-related protein, putative | 1.00 | 3.06 | 2.00 |
| CNH03310 | hypothetical protein | 1.00 | 3.55 | 2.00 |
| CNH01740 | expressed protein | 1.00 | 2.66 | 2.00 |
| CNH02030 | cytochrome b5, putative | -1.00 | 3.94 | -2.00 |
| CNC07140 | unknown | -1.00 | 2.18 | -2.01 |
| CNF02230 | Glutathione S-transferase 6, putative | -1.01 | 2.36 | -2.01 |
| CNC02120 | conserved hypothetical protein | -1.02 | 3.04 | -2.02 |
| CNB00430 | conserved hypothetical protein | -1.02 | 2.35 | -2.03 |
| CNG00860 | mitochondrial processing peptidase beta subunit, mitochondrial precursor (beta-mpp), putative | -1.02 | 2.26 | -2.03 |
| CND03000 | conjugation with cellular fusion-related protein, putative | -1.03 | 2.01 | -2.04 |
| CNC02820 | conserved hypothetical protein | -1.03 | 3.17 | -2.04 |
| CNA06130 | expressed protein | -1.03 | 2.80 | -2.04 |
| CNJ01860 | hypothetical protein | -1.03 | 2.66 | -2.04 |
| CNA04050 | elongation factor 1-gamma (ef-1-gamma), putative | -1.03 | 2.17 | -2.04 |
| CNA06440 | nucleoporin nsp1, putative | -1.03 | 2.78 | -2.05 |
| CNE02810 | tRNA binding protein, putative | -1.03 | 2.99 | -2.05 |
| CNC02680 | conserved hypothetical protein | -1.04 | 2.34 | -2.05 |
| CNA00940 | expressed protein | -1.05 | 2.47 | -2.07 |
| CNA06790 | purine nucleotide biosynthesis-related protein, putative | -1.05 | 3.48 | -2.07 |
| CNN00660 | glucan 1,3 beta-glucosidase protein putative | -1.05 | 2.31 | -2.07 |
| CNI00050 | tRNA dihydrouridine synthase, putative | -1.05 | 2.69 | -2.08 |
| CNM00960 | hypothetical protein | -1.06 | 3.70 | -2.08 |
| CNG03270 | RNA-3-phosphate cyclase, putative | -1.06 | 3.72 | -2.09 |
| CNH02050 | hypothetical protein | -1.06 | 3.25 | -2.09 |
| CNE00920 | conserved hypothetical protein | -1.06 | 2.32 | -2.09 |
| CNI03640 | cell cycle-related protein, putative | -1.06 | 2.09 | -2.09 |
| CNG01310 | arginine-tRNA ligase, putative | -1.06 | 3.01 | -2.09 |
| CNI03200 | hypothetical protein | -1.07 | 2.02 | -2.10 |
| CNE01520 | hypothetical protein | -1.07 | 3.17 | -2.10 |
| CNB03850 | conserved hypothetical protein | -1.08 | 3.74 | -2.11 |
| CNF04580 | conserved hypothetical protein | -1.08 | 2.49 | -2.11 |
| CND06220 | translation elongation factor 2 | -1.08 | 2.40 | -2.12 |
| CNE01000 | GTPase activating protein, putative | -1.08 | 2.88 | -2.12 |
| CNB05560 | conserved hypothetical protein | -1.09 | 2.35 | -2.12 |
| CNC04890 | ornithine decarboxylase, putative | -1.09 | 3.03 | -2.13 |
| CNJ02240 | signal recognition particle protein, putative | -1.09 | 2.31 | -2.13 |
| CNC00090 | conserved hypothetical protein | -1.09 | 3.21 | -2.13 |
| CNH02880 | hypothetical protein | -1.09 | 2.07 | -2.13 |
| CNE01990 | hypothetical protein | -1.09 | 2.56 | -2.13 |
| CNB04640 | rRNA processing-related protein, putative | -1.10 | 3.94 | -2.14 |
| CNB04370 | hypothetical protein | -1.10 | 3.51 | -2.14 |
| CNH01140 | expressed protein | -1.10 | 3.05 | -2.15 |
| CNG00400 | aspartate--tRNA ligase, putative | -1.10 | 3.19 | -2.15 |
| CND02620 | aconitase, putative | -1.10 | 5.53 | -2.15 |
| CNJ00550 | hypothetical protein | -1.10 | 2.35 | -2.15 |
| CNE04840 | conserved hypothetical protein | -1.11 | 2.24 | -2.15 |
| CNC02910 | hypothetical protein | -1.11 | 3.33 | -2.15 |
| CND03820 | myosin I binding protein, putative | -1.11 | 2.22 | -2.15 |
| CNF03750 | DNA-directed RNA polymerase iii 34 kda polypeptide, putative | -1.12 | 2.63 | -2.17 |
| CNA03960 | glyoxal oxidase precursor, putative | -1.12 | 5.17 | -2.17 |
| CNB01740 | hypothetical protein | -1.13 | 3.80 | -2.19 |
| CNC03270 | hypothetical protein | -1.13 | 2.01 | -2.19 |
| CNG03640 | DNA-(apurinic or apyrimidinic site) lyase, putative | -1.14 | 2.54 | -2.20 |
| CNN00480 | chromosome organization and biogenesis -related protein, putative | -1.14 | 2.16 | -2.20 |
| CNA01490 | conserved hypothetical protein | -1.14 | 2.49 | -2.20 |
| CNC04280 | conserved hypothetical protein | -1.14 | 4.44 | -2.20 |
| CNC02570 | hypothetical protein | -1.14 | 2.48 | -2.20 |
| CNF03140 | retrotransposon nucleocapsid protein, putative | -1.14 | 2.79 | -2.20 |
| CNJ02230 | calcineurin A catalytic subunit, putative (NOT CNA1) | -1.14 | 3.75 | -2.20 |
| CNN00350 | glutamate-cysteine ligase, putative | -1.15 | 2.66 | -2.21 |
| CNA07800 | hypothetical protein | -1.15 | 2.82 | -2.21 |
| CNN01920 | unknown | -1.15 | 3.31 | -2.22 |
| CNE04230 | hypothetical protein | -1.15 | 3.73 | -2.22 |
| CNF01120 | hypothetical protein | -1.16 | 2.36 | -2.23 |
| CND00300 | ATP-binding cassette (ABC) transporter, putative | -1.17 | 3.33 | -2.24 |
| CNG04290 | conserved hypothetical protein | -1.17 | 2.35 | -2.25 |
| CNJ00010 | conserved hypothetical protein | -1.17 | 2.27 | -2.25 |
| CNJ02710 | membrane protein, putative | -1.17 | 2.15 | -2.25 |
| CNE03100 | phosphoketolase, putative | -1.17 | 3.17 | -2.25 |
| CNE04920 | hypothetical protein | -1.17 | 3.20 | -2.26 |
| CNH01210 | Pi-transporter A-1, putative | -1.18 | 3.13 | -2.26 |
| CND01010 | hypothetical protein | -1.19 | 2.31 | -2.28 |
| CNC06480 | hypothetical protein | -1.19 | 2.50 | -2.28 |
| CNG03480 | unknown | -1.21 | 5.53 | -2.31 |
| CNG00290 | hypothetical protein | -1.21 | 3.26 | -2.32 |
| CNA05600 | catalase A, putative | -1.21 | 3.89 | -2.32 |
| CNH00330 | conserved hypothetical protein | -1.22 | 4.49 | -2.33 |
| CNG03340 | conserved hypothetical protein | -1.22 | 2.01 | -2.34 |
| CNA06420 | expressed protein | -1.23 | 2.65 | -2.34 |
| CNI01790 | conserved hypothetical protein | -1.23 | 3.17 | -2.35 |
| CNE00190 | structural constituent of nuclear pore, putative | -1.24 | 2.60 | -2.37 |
| CNG00780 | cytoplasm protein, putative | -1.24 | 3.33 | -2.37 |
| CNG04380 | WD repeat protein, putative | -1.25 | 2.69 | -2.37 |
| CNG03510 | RNA lariat debranching enzyme, putative | -1.25 | 3.95 | -2.38 |
| CNG02210 | cytochrome-c oxidase, putative | -1.25 | 5.16 | -2.39 |
| CNF01610 | chitin synthase-related | -1.26 | 2.45 | -2.39 |
| CNL04280 | conserved hypothetical protein | -1.26 | 3.81 | -2.39 |
| CNE02800 | conserved hypothetical protein | -1.27 | 2.89 | -2.41 |
| CNF02710 | Rho GTPase activator, putative | -1.28 | 2.07 | -2.42 |
| CNL04780 | carbonic anhydrase protein, putative | -1.29 | 2.01 | -2.44 |
| CNM02530 | expressed protein | -1.30 | 2.46 | -2.46 |
| CNK00390 | sodium-hydrogen antiporter | -1.30 | 4.95 | -2.47 |
| CNE01150 | NADH-ubiquinone oxidoreductase 12 kda subunit, mitochondrial precursor, putative | -1.31 | 3.48 | -2.47 |
| CNH02250 | hypothetical protein | -1.32 | 4.59 | -2.50 |
| CNI03040 | expressed protein | -1.32 | 2.61 | -2.50 |
| CNA02900 | hypothetical protein | -1.32 | 4.27 | -2.50 |
| CNF03140 | retrotransposon nucleocapsid protein, putative | -1.33 | 2.13 | -2.51 |
| CNI02850 | ATP-dependent RNA helicase, putative | -1.33 | 2.44 | -2.51 |
| CNA01350 | splicing factor Prp8, putative | -1.33 | 2.87 | -2.51 |
| CNC01530 | fermentation-related protein, putative | -1.34 | 2.60 | -2.53 |
| CNL03700 | glutamate decarboxylase, putative | -1.34 | 3.05 | -2.53 |
| CNH01530 | dihydroxy-acid dehydratase, putative | -1.35 | 3.33 | -2.54 |
| CNH01030 | NADH dehydrogenase 10.5K chain, putative | -1.35 | 3.03 | -2.55 |
| CNB03900 | transcriptional regulatory protein, putative | -1.35 | 2.88 | -2.55 |
| CNJ01380 | midasin, putative | -1.35 | 2.58 | -2.55 |
| CNL05500 | sulfite reductase, putative | -1.36 | 4.46 | -2.56 |
| CNM01030 | tricarboxylate carrier, putative | -1.36 | 2.01 | -2.56 |
| CNC02250 | hydroxymethylbilane synthase, putative | -1.36 | 4.72 | -2.56 |
| CNB02300 | integral to membrane protein, putative | -1.36 | 2.66 | -2.57 |
| CNA01950 | expressed protein | -1.37 | 4.47 | -2.58 |
| CNA04260 | hypothetical protein | -1.37 | 2.84 | -2.59 |
| CND03540 | DNA-dependent RNA polymerase II RPB140, putative | -1.38 | 3.82 | -2.60 |
| CNL04990 | hypothetical protein | -1.39 | 3.72 | -2.62 |
| CNA07550 | regulation of transcription from Pol II promoter-related protein, putative | -1.41 | 2.27 | -2.66 |
| CNK02510 | uroporphyrinogen-III synthase, putative | -1.41 | 2.93 | -2.66 |
| CNE01200 | rRNA processing-related protein, putative | -1.42 | 3.88 | -2.67 |
| CNA05690 | hypothetical protein | -1.42 | 3.18 | -2.68 |
| CNF00890 | importin beta-4 subunit, putative | -1.42 | 2.86 | -2.68 |
| CNH02740 | ubiquinol-cytochrome c reductase complex 7.3 kda protein, putative | -1.43 | 3.62 | -2.70 |
| CNJ02740 | vacuole protein, putative | -1.44 | 2.29 | -2.71 |
| CNJ01450 | unknown | -1.44 | 3.62 | -2.71 |
| CNA03960 | glyoxal oxidase precursor, putative | -1.45 | 5.54 | -2.73 |
| CNL04470 | ubiquinol-cytochrome C reductase complex core protein 2 precursor, putative | -1.46 | 3.98 | -2.74 |
| CND05120 | 3-deoxy-7-phosphoheptulonate synthase, putative | -1.46 | 3.10 | -2.76 |
| CNA07330 | hypothetical protein | -1.47 | 6.05 | -2.78 |
| CNL03740 | catalase, putative | -1.48 | 5.51 | -2.79 |
| CNH03630 | conserved hypothetical protein | -1.49 | 2.28 | -2.80 |
| CNE03290 | eukaryotic translation initiation factor 3 110 kda subunit (eif3 p110), putative | -1.49 | 2.61 | -2.81 |
| CNH02180 | hypothetical protein | -1.49 | 2.17 | -2.82 |
| CNF03560 | hypothetical protein | -1.50 | 2.65 | -2.83 |
| CNG03860 | nucleus protein, putative | -1.51 | 2.70 | -2.85 |
| CND01250 | hypothetical protein | -1.52 | 5.04 | -2.87 |
| CNC04490 | hypothetical protein | -1.52 | 4.55 | -2.87 |
| CNI03950 | expressed protein | -1.53 | 2.15 | -2.88 |
| CNJ03280 | tRNA methyltransferase, putative | -1.54 | 2.89 | -2.92 |
| CND04690 | transporter, putative | -1.55 | 3.86 | -2.92 |
| CNA05710 | conserved hypothetical protein | -1.56 | 2.91 | -2.95 |
| CNH02740 | ubiquinol-cytochrome c reductase complex 7.3 kda protein, putative | -1.57 | 5.28 | -2.96 |
| CNF00920 | mannosyltransferase, putative | -1.57 | 2.38 | -2.97 |
| CNG03480 | unknown | -1.57 | 3.38 | -2.97 |
| CNB04290 | nucleic acid binding protein, putative | -1.58 | 2.60 | -2.99 |
| CNI03610 | lactoylglutathione lyase, putative | -1.58 | 3.76 | -2.99 |
| CNC07080 | hypothetical protein | -1.61 | 2.64 | -3.06 |
| CNH00900 | conserved hypothetical protein | -1.62 | 3.40 | -3.07 |
| CNF00970 | cytochrome c heme lyase (cchl), putative | -1.62 | 4.08 | -3.08 |
| CNB01510 | hypothetical protein | -1.64 | 3.25 | -3.12 |
| CNM01610 | hypothetical protein | -1.66 | 2.47 | -3.16 |
| CNB05400 | hypothetical protein | -1.67 | 4.84 | -3.17 |
| CNM01490 | hypothetical protein | -1.67 | 3.16 | -3.19 |
| CNC01790 | conserved hypothetical protein | -1.68 | 3.12 | -3.21 |
| CNF03780 | malate dehydrogenase, putative | -1.70 | 3.44 | -3.25 |
| CNG00530 | conserved hypothetical protein | -1.70 | 5.53 | -3.26 |
| CND04430 | ubiquinol-cytochrome-c reductase, putative | -1.71 | 5.05 | -3.27 |
| CNC03380 | expressed protein | -1.73 | 4.31 | -3.31 |
| CND00550 | glucosidase, putative | -1.73 | 2.46 | -3.33 |
| CNC03390 | conserved hypothetical protein | -1.80 | 5.09 | -3.49 |
| CNN01400 | copper-exporting ATPase, putative | -1.81 | 2.10 | -3.50 |
| CNJ01450 | unknown | -1.81 | 4.12 | -3.51 |
| CNK00580 | homoaconitate hydratase, putative | -1.82 | 3.24 | -3.52 |
| CNA06940 | hypothetical protein | -1.83 | 4.07 | -3.56 |
| CNK00570 | expressed protein | -1.85 | 3.45 | -3.59 |
| CNE03240 | chitin synthase, putative | -1.85 | 4.02 | -3.61 |
| CNJ00510 | hypothetical protein | -1.86 | 4.34 | -3.63 |
| CNE02290 | arginine N-methyltransferase 3, putative | -1.87 | 3.74 | -3.66 |
| CNM01400 | conserved hypothetical protein | -1.88 | 3.37 | -3.67 |
| CNA03530 | succinate dehydrogenase (ubiquinone), putative | -1.89 | 3.96 | -3.71 |
| CNG04420 | alpha-1,3-glucan synthase, putative | -1.92 | 4.75 | -3.78 |
| CNA05700 | expressed protein | -1.92 | 4.46 | -3.78 |
| CNA00150 | holocytochrome-c synthase, putative | -1.95 | 3.68 | -3.85 |
| CNJ01790 | hypothetical protein | -1.97 | 3.87 | -3.92 |
| CND06160 | conserved hypothetical protein | -2.02 | 2.36 | -4.06 |
| CNH02730 | NADH-ubiquinone oxidoreductase, putative | -2.05 | 3.62 | -4.15 |
| CNK03030 | hypothetical protein | -2.08 | 2.32 | -4.23 |
| CNA05580 | conserved hypothetical protein | -2.12 | 5.80 | -4.33 |
| CNJ00560 | conserved hypothetical protein | -2.12 | 2.32 | -4.33 |
| CNL06640 | phospho-2-dehydro-3-deoxyheptonate aldolase, putative | -2.12 | 2.95 | -4.35 |
| CNG03580 | conserved hypothetical protein | -2.15 | 4.48 | -4.43 |
| CNF00630 | electron transporter, transferring electrons within CoQH2-cytochrome c reductase complex, putative | -2.15 | 3.45 | -4.44 |
| CND03800 | GAMM1 protein, putative | -2.18 | 4.60 | -4.52 |
| CNL05770 | conserved hypothetical protein | -2.18 | 2.76 | -4.53 |
| CNA05180 | expressed protein | -2.19 | 4.05 | -4.55 |
| CNK01280 | expressed protein | -2.22 | 3.44 | -4.67 |
| CNB01310 | NADH-ubiquinone oxidoreductase, putative | -2.26 | 3.76 | -4.80 |
| CNB00170 | expressed protein | -2.33 | 3.10 | -5.02 |
| CNA06950 | electron carrier, putative | -2.37 | 2.89 | -5.18 |
| CNM01810 | conserved hypothetical protein | -2.47 | 4.13 | -5.55 |
| CNA07540 | 88 kDa immunoreactive mannoprotein MP88, putative | -2.58 | 2.91 | -5.98 |
| CNE05040 | glyoxal oxidase precursor, putative | -2.60 | 3.65 | -6.08 |
| CNM01390 | conserved hypothetical protein | -2.62 | 4.11 | -6.15 |
| CNM02270 | conserved hypothetical protein | -2.67 | 3.48 | -6.36 |
| CNH01720 | hypothetical protein | -2.70 | 4.20 | -6.51 |
| CND03490 | chitin deacetylase-like mannoprotein MP98 | -2.70 | 4.63 | -6.52 |
| CNE03960 | conserved hypothetical protein | -2.73 | 3.68 | -6.65 |
| CND04070 | NADH-ubiquinone oxidoreductase 51 kDa subunit, putative | -2.74 | 3.75 | -6.67 |
| CNL03730 | glucose-methanol-choline (GMC) oxidoreductase, putative | -2.76 | 3.40 | -6.79 |
| CND00390 | multidrug efflux pump, putative | -2.87 | 2.84 | -7.31 |
| CNA02980 | conserved hypothetical protein | -2.89 | 6.08 | -7.42 |
| CNC07090 | conserved hypothetical protein | -2.91 | 4.31 | -7.51 |
| CNA04420 | oxidoreductase, putative | -2.98 | 2.99 | -7.88 |
| CNC06180 | conserved hypothetical protein | -3.06 | 4.40 | -8.34 |
| CNH01100 | NADPH dehydrogenase, putative | -3.10 | 4.32 | -8.57 |
| CNM02030 | conserved hypothetical protein | -3.38 | 2.16 | -10.44 |
| CNL06730 | conserved hypothetical protein | -3.48 | 4.01 | -11.13 |
